# Genome–metabolite associations revealed low heritability, high genetic complexity, and causal relations for leaf metabolites in winter wheat (*Triticum aestivum*)

**DOI:** 10.1093/jxb/erw441

**Published:** 2016-12-22

**Authors:** Andrea Matros, Guozheng Liu, Anja Hartmann, Yong Jiang, Yusheng Zhao, Huange Wang, Erhard Ebmeyer, Viktor Korzun, Ralf Schachschneider, Ebrahim Kazman, Johannes Schacht, Friedrich Longin, Jochen Christoph Reif, Hans-Peter Mock

**Affiliations:** 1 Department of Physiology and Cell Biology, Applied Biochemistry, Leibniz Institute of Plant Genetics and Crop Plant Research, 06466 Gatersleben, Germany; 2 Department of Breeding Research, Quantitative Genetics, Leibniz Institute of Plant Genetics and Crop Plant Research, 06466 Gatersleben, Germany; 3 Department of Physiology and Cell Biology, Molecular Plant Nutrition, Leibniz Institute of Plant Genetics and Crop Plant Research, 06466 Gatersleben, Germany; 4 Biometris, Department of Plant Sciences, Wageningen University, 6708 PB Wageningen, The Netherlands; 5 KWS LOCHOW GmbH, 29296 Bergen, Germany; 6 Nordsaat Saatzuchtgesellschaft mbH, 38895 Langenstein, Germany; 7 Lantmännen SW Seed Hadmersleben GmbH, 39398 Hadmersleben, Germany; 8 Limagrain GmbH, 31226 Peine-Rosenthal, Germany; 9 University of Hohenheim, State Plant Breeding Institute, 70599 Stuttgart, Germany

**Keywords:** Genome-wide association, metabolomics, mQTL, network analysis, Triticum aestivum, winter wheat

## Abstract

We investigated associations between the metabolic phenotype, consisting of quantitative data of 76 metabolites from 135 contrasting winter wheat (*Triticum aestivum*) lines, and 17 372 single nucleotide polymorphism (SNP) markers. Metabolite profiles were generated from flag leaves of plants from three different environments, with average repeatabilities of 0.5–0.6. The average heritability of 0.25 was unaffected by the heading date. Correlations among metabolites reflected their functional grouping, highlighting the strict coordination of various routes of the citric acid cycle. Genome-wide association studies identified significant associations for six metabolic traits, namely oxalic acid, ornithine, L-arginine, pentose alcohol III, L-tyrosine, and a sugar oligomer (oligo II), with between one and 17 associated SNPs. Notable associations with genes regulating transcription or translation explained between 2.8% and 32.5% of the genotypic variance (*p*_*_G_*_). Further candidate genes comprised metabolite carriers (*p*_*_G_*_ 32.5–38.1%), regulatory proteins (*p*_*_G_*_ 0.3–11.1%), and metabolic enzymes (*p*_*_G_*_ 2.5–32.5%). The combinatorial use of genomic and metabolic data to construct partially directed networks revealed causal inferences in the correlated metabolite traits and associated SNPs. The evaluated causal relationships will provide a basis for predicting the effects of genetic interferences on groups of correlated metabolic traits, and thus on specific metabolic phenotypes.

## Highlight

The associations and causal relations revealed between 76 metabolites from 135 winter wheat lines and 17 372 SNP markers allow the genetic interventions on sets of metabolites to be dissected.

## Introduction

Metabolomics is complementing the molecular breeding toolbox, facilitating the prediction of a number of important agronomic traits (Saito and Matsuda, 2010). Metabolite signatures are associated with plant growth (Meyer *et al.*, 2007) and have been used to predict complex traits such as biomass (Riedelsheimer *et al.*, 2012a) and grain yield (Xu *et al.*, 2016). Furthermore, metabolites play key roles in plant defence against abiotic and biotic stresses (Buchanan *et al.*, 2015). Hence, metabolite profiling has become a central component in obtaining a more holistic view of the effects of disease and abiotic stress on plant performance (Moco *et al.*, 2007; Deborde *et al.*, 2011; Kueger *et al.*, 2012). This also holds true for fruit and seed traits in tomato (*Solanum lycopersicum*) (Schauer *et al.*, 2006; Toubiana *et al.*, 2012; Sauvage *et al.*, 2014). The increasing role of metabolites as indirect traits in plant breeding requires a greater understand of the details of their genetic architecture.

Linkage mapping has been applied to dissect the genetic architecture of metabolite abundance in *Arabidopsis thaliana* (Keurentjes *et al.*, 2006; Lisec *et al.*, 2009) and rice (*Oryza sativa*) (Gong *et al.*, 2013). Several quantitative trait loci (QTL) have been detected that control metabolite abundance; they are referred to as metabolomic QTL (mQTL). It is believed that genetic variation in metabolite abundance could be important in the adaptations to specific environmental conditions that allow particular genotypes to grow. mQTL analyses have helped to uncover co-regulated compounds and metabolic pathways, such as for glucosinolate (Keurentjes *et al.*, 2006) and flavonoid (Gong *et al.*, 2013) biosynthesis. Variation in the abundance of metabolites in the rice grain is largely determined by mQTL acting in concert with various regulatory factors (Matsuda *et al.*, 2012). Several mQTL have been identified in maize (*Zea mays*), both in the grain (Zheng *et al.*, 2008; Yan *et al.*, 2011; Wen *et al.*, 2014) and in the leaf (Riedelsheimer *et al.*, 2012*b*; Wen *et al.*, 2015). The above-outlined genome-wide mapping studies revealed several putative mQTL, suggesting that metabolite abundance has a complex genetic architecture in plants.

Wheat (*Triticum aestivum* L.) is one of the world’s most important crops, providing 20% of the total calories for the world’s population. Despite its economic relevance, only one study based on 233 doubled haploid lines has performed genome-wide mQTL mapping (Hill *et al.*, 2015). This QTL study validated the utility of combining agronomic and metabolomics traits as an approach for identifying potential trait enhancement targets for breeding.

Here, an attempt has been made to associate the varied abundance of a set of 76 leaf metabolites with allelic status at a large number of genetic marker loci. The analysis was based on a panel of 135 European elite winter wheat lines, grown in three different environments. Some of the mQTL identified have been contextualized by applying a partial directed network analysis.

## Materials and methods

### Plant material and harvest

The genetic material consisted of 135 elite winter wheat lines adapted to Central Europe (Zhao *et al.*, 2015). All genotypes were evaluated in replicated grain yield trials at three locations (Böhnshausen, Hadmersleben, and Hohenheim) in Germany in 2012 (Longin *et al.*, 2013). Lines were grown in an incomplete block design with two replicates at each location. For each line, 10 flag leaf samples were randomly selected per replicate and location at the time when >60% of the lines had reached BBCH-69 (Lancashire *et al.*, 1991). All plots were sampled from 9 to 11 am within 120 min as outlined in detail by Zhao *et al.* (2015). Flag leaf samples were snap-frozen in liquid nitrogen, freeze-dried, and powdered in an MM200 ball mill (Retsch, Haan, Germany).

### Metabolite profiling

A mixed sample was assembled composed of equal amounts from each individual sample in order to capture systematic shifts during extraction and measurement. The mixed sample was processed in the same way as all other samples and its data were used to normalize for shifts during metabolite profile analysis across the data set for all three locations. All analyses were performed in duplicate. Polar metabolites were extracted twice with 500 μl of 70% methanol per 20 mg dry weight (DW), with 50 µM ^13^C6/D7 glucose as an internal standard (Sigma-Aldrich, Munich, Germany). The suspension was centrifuged (20000 *g*, 4°C, 20 min) and the two supernatants pooled. For phase separation, 1000 μl of water and 500 μl of chloroform were added. After centrifugation (20000 *g*, 4°C, 10 min), 30 µl of the upper phase was transferred to a glass vial and dried in a vacuum centrifuge.

Derivatization was performed using a MultiPurposeSampler (Gerstel, Mülheim an der Ruhr, Germany) connected to a GC-MS system by first adding 40 μl of methoxyamine hydrochloride (20 mg/ml in pyridine) for 2 h at 37°C, and then adding 40 μl of N-methyl-N-(trimethylsilyl) trifluoroacetamide containing the alkane retention time standard (C7-C30, 10 μg/μl, Sigma-Aldrich, Munich, Germany) for 30 min at 37°C (Lisec *et al.*, 2006). The measuring device was an Agilent A7890 gas chromatograph (Böblingen, Germany) coupled to a Waters GCT Premier time-of-flight mass spectrometer (Milford, MA, USA). A 1 μL derivatized sample was injected in splitless mode at 240°C, and passed through a 30 m Rxi®-5Sil MS column (internal diameter 0.25 mm, and 0.25 μm film thickness) with a 5 m Integra-Guard® column (Restek, Bad Homburg, Germany) using the following parameters: 3 min at 80°C, followed by +5°C/min up to 300°C. Data were acquired with the MassLynx 4.1 software (Waters), capturing 10 spectra per second in the *m/z* range of 65–650 in centroid mode and applying the dynamic enhancement (DRE) mode. Compound annotation was performed by running authentic standards, determining retention indices and specific fragment spectra, and by comparing to information in the NIST database implemented in MassLynx 4.1 software and the Golm Metabolome Database (http://gmd.mpimp-golm.mpg.de/, last accessed 23 November 2016). Data processing and peak integration were performed using the QuanLynx tool implemented in MassLynx 4.1 software, utilizing specific masses for metabolite quantification (Supplementary Table S1 at *JXB* online).

### Metabolic data analyses

The flexible Box–Cox power transformation was applied to achieve homoscedasticity of the residuals of metabolites (Piepho, 2009). Of the 85 metabolites assayed, 76 metabolites were retained after quality assessment (Supplementary Fig. S1 at *JXB* online) as outlined in detail by Anscombe and Tukey (1963). The adjusted entry means were estimated for the genome-wide association study (GWAS) (Zhao *et al.*, 2014). A one-step model was used to estimate the genetic variance components of lines and the variance of genotype × environment interactions. The significance of variance component estimates was tested by comparing models using likelihood ratio tests in which halved *P* values were used as an approximation (Stram and Lee, 1994). Using the variance components we estimated the heritability on an entry-mean basis. We assumed fixed genetic effects and estimated the best linear unbiased estimates (BLUEs) of lines across environments. The metabolite abundances were analysed with the software package ASRemL-R 3.0 (Butler *et al.*, 2009). Pairwise correlations between the abundances of the 76 metabolites were revealed by applying the average linkage clustering method, based on Pearson correlation coefficients. Visualization of abundant metabolite profiles in the various lines, and an integrated visualization of highly correlated metabolites in the context of metabolic pathways and networks, were based on the VANTED framework (http://www.vanted.org, last accessed 23 November 2016; Rohn *et al.*, 2012).

### Genotypic data generation

Fingerprinting of DNA from all genotypes was performed using a 90k SNP array using the Illumina Infinium assay (Gowda *et al.*, 2014). We discarded loci which were monomorphic, had missing values of >5%, heterozygosity of >5% in inbred lines, or for which a minor allele frequency of <5% applied. After applying these quality controls, 17372 SNP markers remained for GWAS. Genomic positions were known for 14298 out of these 17372 SNP. Blast searches for candidate genes were performed against the IWGSCv2.2 database (http://pgsb.helmholtz-muenchen.de/plant/search.jsp, last accessed 23 November 2016; Nussbaumer *et al.* (2013)).

### Genome-wide association mapping

Adjusted entry means of metabolic traits of each environment were used for genome-wide association mapping using a linear mixed model. Correction for population stratification was implemented using a kinship matrix (Jiang *et al.*, 2015). Marker–trait association scans were conducted to detect main-effect mQTL using the following model:

Y=Xβ+Ss+(S⊗X)m+Zu+e,

where *Y* stands for the adjusted entry means of the 135 genotypes of each environment, β is a vector of environment effects, *s* is a vector of SNP effects, *m* is a vector of SNP × environment interaction effects, *u* is a vector of polygene background effects, and *e* is a vector of residual effects. *X*, *S*, S⊗X, and *Z* are incidence matrices relating *Y* to β, *s*, *m*, and *u*. *s* was treated as a fixed effect and β, *m*, and *u* as random effects.

The significance of marker–trait associations was tested based on the Wald F statistic. The significance of SNP × environment interaction effects was determined by a likelihood ratio test (Stram and Lee, 1994). A procedure to control the false discovery rate (FDR) (Benjamini and Hochberg, 1995) was applied to correct for multiple testing at a significance level of *P* < 0.20. The proportion of the phenotypic variance explained by a single QTL (*R*^*2*^) and by all mQTL (adjusted *R*^*2*^) was estimated using a standard multiple regression approach (Utz *et al.*, 2000). The proportion of the genotypic variance explained by a single mQTL and by all mQTL (*p*_*G*_) was determined as a proportion of the explained phenotypic variance standardized by broad-sense heritability. Association mapping analyses were performed using the ASReml-R 3.0 software (Butler *et al.*, 2009).

### Inferring causal phenotype networks

Causal phenotype networks among the metabolomic data were inferred using the heuristic QTL+phenotype supervised orientation (QPSO) search algorithm (Wang and van Eeuwijk, 2014). In the first step, undirected networks among all metabolites were pre-learnt using the PC-skeleton algorithm (Spirtes *et al.*, 2000) implemented with the R package pcalg (Kalisch *et al.*, 2012). In the second step, significant mQTL (*P* < 0.1, FDR correction) were added into the undirected network and the QPSO algorithm applied.

## Results

### Metabolite profiles are complex heritable traits with pronounced genotype × environment interaction

**Fig. 1.**
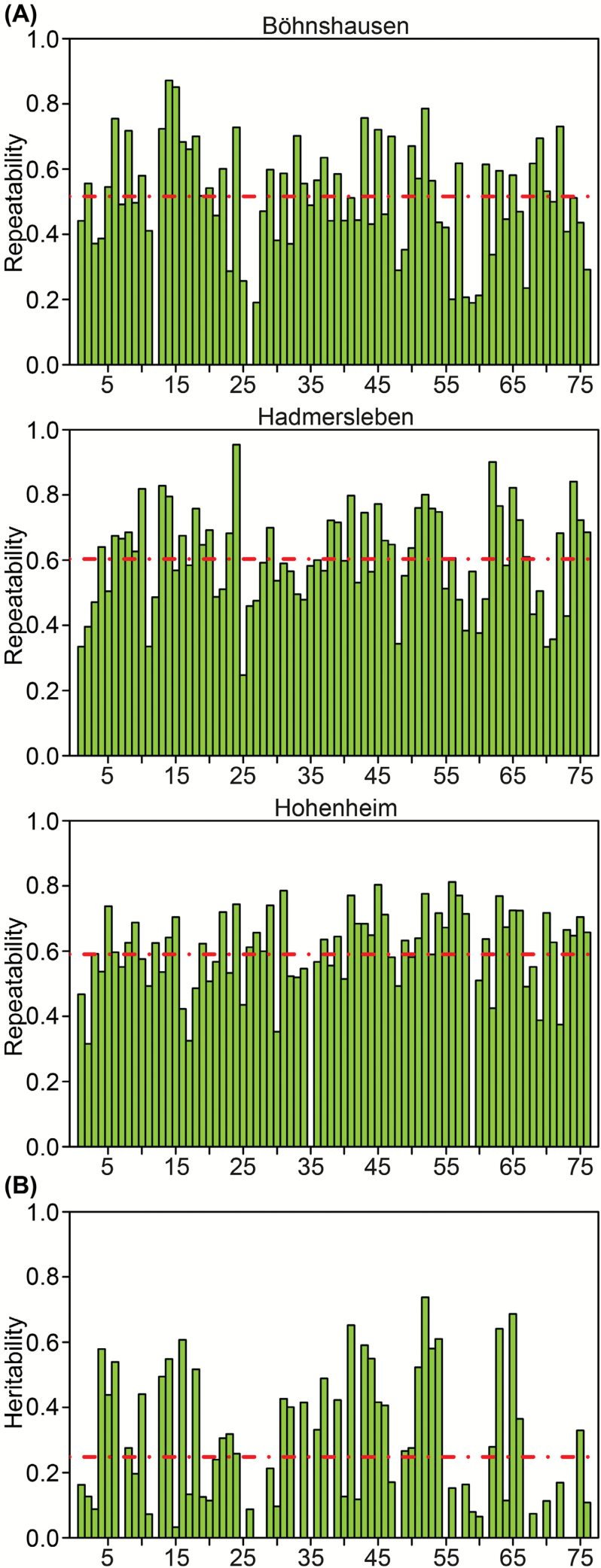
(**A**) Reproducibility of metabolite abundances across the three field sites. The mean values, with 0.52 for Böhnshausen, 0.60 for Hadmersleben, and 0.59 for Hohenheim are indicated as dashed lines. (**B**) The heritability of abundance of the 76 metabolites across the three locations Hadmersleben, Böhnshausen, and Hohenheim. The mean value (0.25) is indicated as a dashed line.

**Fig. 2.**
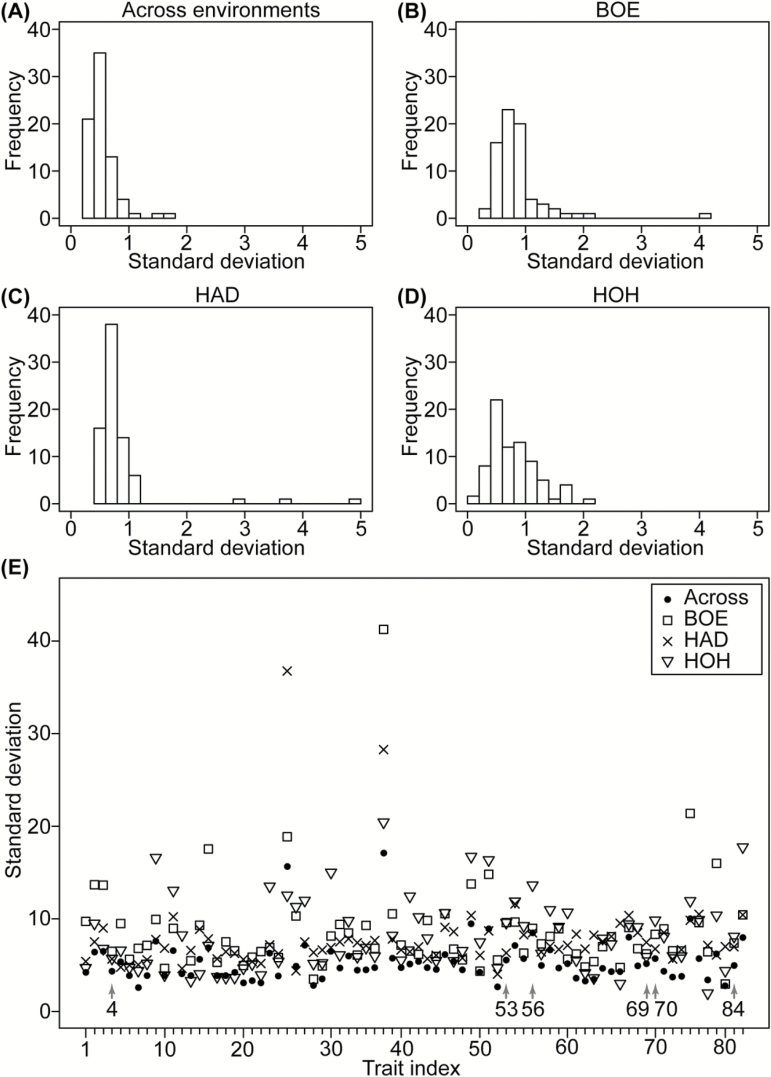
Variation for standard deviations of metabolite profiles across and within environments. (**A**) Histogram for BLUEs across the three environments. Histograms for BLUEs within (**B**) Böhnshausen, (**C**) Hadmersleben, and (**D**) Hohenheim. (**E**) Individual variation of standard deviation for the investigated metabolites, red arrows indicate the six significantly associated metabolic traits.

A total of 85 metabolites were recognized, but after quality evaluation, only 76 were retained for further analyses (Supplementary Tables S1 and S2; Fig. S2 at *JXB* online). The level of repeatability of metabolite abundance did not vary between the three sites, with a mean value of 0.52 at Böhnshausen, 0.60 at Hadmersleben, and 0.59 at Hohenheim (Fig. 1A). Furthermore, we observed no differences in the standard deviations of the genotypes across the three environments (Fig. 2). These findings indicate that the sampling and analytical strategies were appropriate and resulted in high-quality data regarding metabolite abundance.

The analyses of variance revealed a strong linear effect of the environments on the metabolite profiles (Supplementary Table S2). Individual metabolite profiles varied substantially across the three sites (Supplementary Figs S3 and S4 at *JXB* online), resulting in variances of genotypes that were on average only half the size of the variances of genotype × location interactions. As a result, the broad-sense heritabilities were low, with a global mean of 0.25 (Fig. 1B). In all, 24 of the metabolite abundances had a heritability of >0.4 and variances of genotypes which were significantly (*P* < 0.001) larger than zero (Supplementary Table S2). For 17 metabolite abundances, non-significant (*P* > 0.9) variation of genotypic effects was observed.

To evaluate the influence of the genetic differences in the 135 lines with respect to their developmental stage at the time the flag leaves were sampled, we estimated heritabilities for subgroups of early-, intermediate-, and late-maturing lines. The heritability estimates were similar for all three classes of lines (early 0.29, intermediate 0.23, late 0.21) (Supplementary Fig. S5 at *JXB* online). This implies that differences in heading date did not lead to a decrease in the heritability of flag leaf metabolite profiles in the total population of 135 lines.

### Correlations among metabolites highlight the various routes of the citric acid cycle

**Fig. 3.**
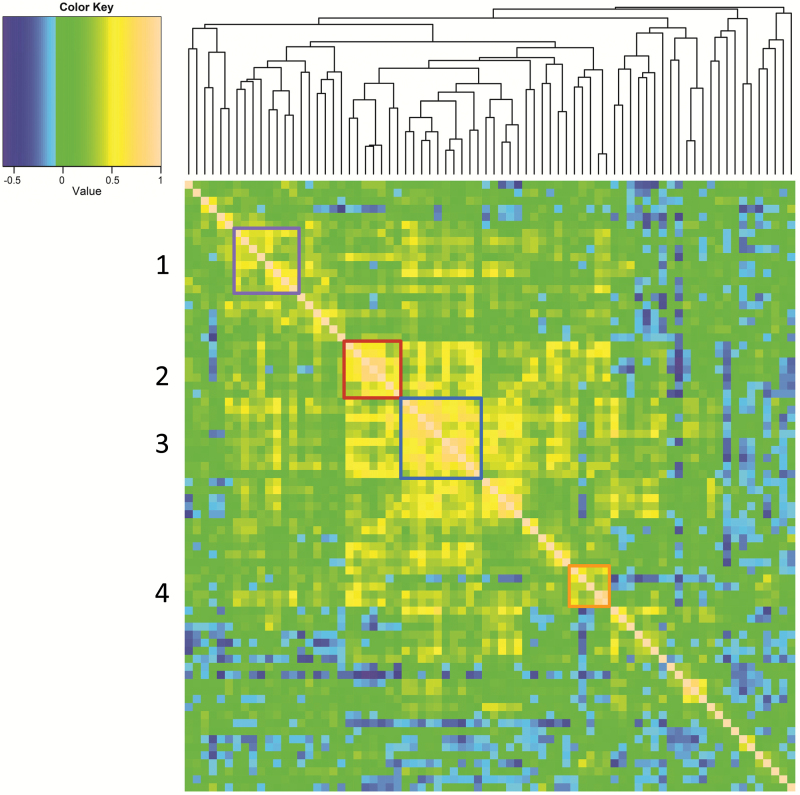
Correlation pattern among metabolites. Pairwise Pearson correlations are shown in a heat map representation, whereas metabolites are sorted according to correlation-based hierarchical cluster analysis. High positive correlations are represented by earth tone squares and negative ones by blue squares. The raw data are presented in Supplementary Table S4, and annotation of the cluster tree arms is shown in Supplementary Fig. S6.

**Fig. 4.**
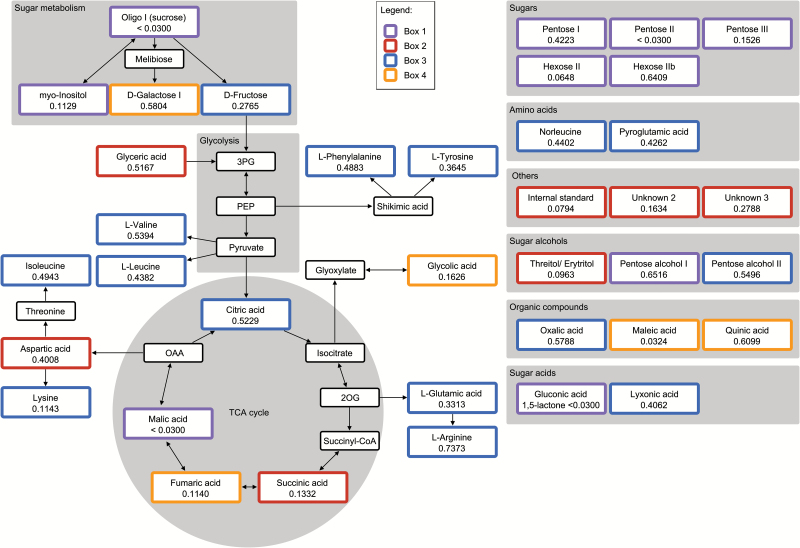
Pathways involving the strongly correlated metabolite pairs indicated in Fig. 3. The most prominent groups represent elements of the citric acid cycle. Unmapped metabolites have been sorted into compound groups and are shown in the right panel. Frame colours refer to box 1 (violet), box 2 (red), box 3 (blue), and box 4 (orange). The heritability of abundance of the presented metabolites across the three locations Hadmersleben, Böhnshausen, and Hohenheim is shown within the relevant boxes.

Out of the 2850 pairwise comparisons of metabolites, 794 (27.86%) were highly significantly correlated (*P* < 0.001), 1124 (39.44%) were moderately significantly correlated (*P* < 0.01), and 1462 (51.30%) were just significantly correlated (*P* < 0.05) (Supplementary Table S3 at *JXB* online). A number of regions with tightly correlated metabolites were identified, often reflecting functional relationships. Some examples are highlighted in Fig. 3, with detailed annotations given in Supplementary Fig. S6 at *JXB* online. One such group comprised sugars and sugar alcohols (box 1), one was a set of organic acids associated with the citric acid cycle (boxes 2 and 4), and another included various small organic acids and amino acids (box 3). Pathway analysis showed that metabolites representing the main entry points of glycolysis and the citric acid cycle clustered within box 1 (Fig. 4, shown in violet); a second group represented intermediates of amino acid and nucleic acid biosynthesis (box 2, red). The large metabolite group clustered within box 3 (blue) comprised amino acids involved in the citric acid cycle, together with key metabolites such as D-fructose and citric acid. Small organic acids related to the glycolate cycle and photorespiration clustered into box 4 (orange), implying a degree of joint regulation between photosynthesis and the citric acid/ glycolate cycles.

### Metabolomics and genomic diversity

We estimated distance matrices among the 135 lines based on the metabolite abundances or the genomic profiles. These matrices were only weakly associated, with a correlation coefficient of r = 0.02 (*P* < 0.34). A cluster analysis based on the genome-wide SNP marker data revealed the absence of a major population structure but the presence of a family structure among the 135 parental lines (Supplementary Fig. S7 at *JXB* online). Therefore, a linear mixed model was applied for the GWAS to correct for relatedness, modelling a kinship matrix.

### GWA mapping revealed associations for six metabolic traits

**Fig. 5.**
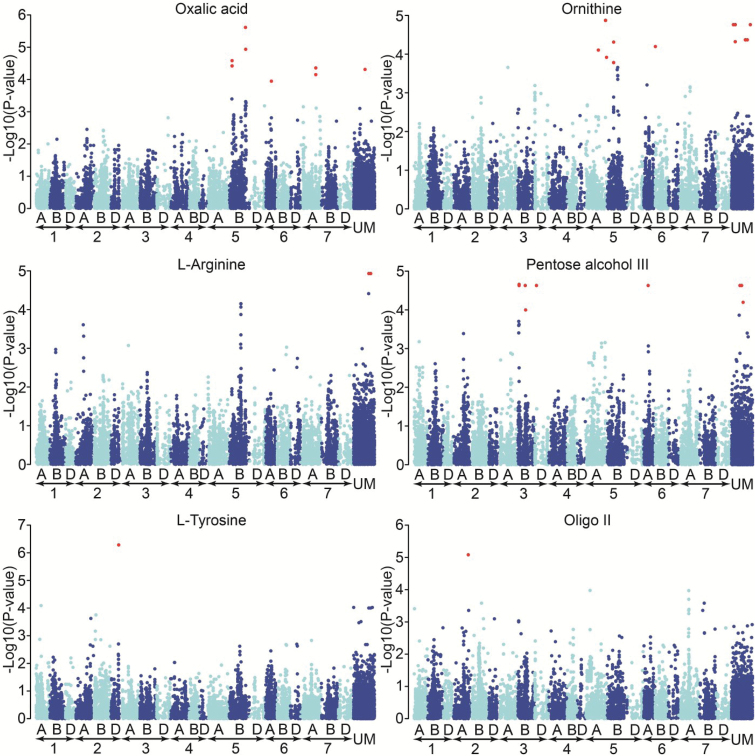
Manhattan plots displaying the GWA mapping analysis for metabolites generating a significant association signal. Significant *P* values (0.2 after FDR correction) are shown in red.

The GWA scan was applied to the 34 metabolites for which abundance was associated with a significant degree of genotypic variance. The resulting six significant SNP–metabolite abundance associations involved 40 SNPs and oxalic acid, ornithine, L-arginine, pentose alcohol III, L-tyrosine, and oligo II (Table 1 and Fig. 5). Interestingly, none of the 40 SNPs exhibited significant interaction effects with the environments (Table 1).

**Table 1.** Summary of GWA mapping

Metabolite	*H* ^2^	Locus	Chromosome position	*P*_SNP_	*P*_SNP×Environment_	*R* ^2^	*p*_G_	Gene ID or accession	Splice variants	Candidate gene product	Variation type
Oxalic acid	0.578	IWB8786*	5B 548.67cM	2.43E-06	1	11.88	20.55	no match		Unknown	
		IWB4387	5B 558.07cM	1.15E-05	1	0.06	0.10	no match		Unknown	
		IWB14354*	7A 532.61cM	4.40E-05	1	6.60	11.42	Traes_7DL_98245621B.2	1	Transcription elongation factor (TFIIS)	N
		IWB35367*	Not Mapped	4.88E-05	1	11.11	19.22	Traes_5BS_EA4A42ADB.1	1	GTP-binding protein	S
		IWB47615*	5B 92.03cM	3.78E-05	1	1.79	3.10	no match		Unknown	
		IWA3211	5B 92.03cM	3.78E-05	1	s.a.	s.a.	no match		Unknown	
		IWA6946	5B 92.03cM	3.78E-05	1	s.a.	s.a.	no match		Unknown	
		IWA6947	5B 92.03cM	2.61E-05	1	0.13	0.22	no match		Unknown	
		IWB29877	7A 532.61cM	7.10E-05	1	0.09	0.16	Traes_7AL_968CDEFCB.2	1	Transcription elongation factor (TFIIS)	N
		IWB33544*	6A 190.27cM	1.14E-04	1	6.72	11.63	Traes_6BS_60D771A56.1	1	Galactose oxidase/kelch repeat protein	S
Ornithine	0.27	IWB4446*	5A 703.91cM	1.34E-05	1	10.28	38.07	Traes_5AL_F49663738.2	1	Hexose carrier protein HEX6	N
		IWB6885*	Not Mapped	1.74E-05	1	12.16	45.04	no match		Unknown	
		IWB8628	Not Mapped	1.74E-05	1	s.a.	s.a.	no match		Unknown	
		IWB8637	Not Mapped	1.74E-05	1	s.a.	s.a.	no match		Unknown	
		IWB60850	5A 709.71cM	1.74E-05	1	s.a.	s.a.	Traes_4DL_FA7457EA3.1	1	Hexose carrier protein HEX6	N
		IWB75174	Not Mapped	1.74E-05	1	s.a.	s.a.	no match		Unknown	
		IWB3615*	5B 222.57cM	4.90E-05	1	5.48	20.30	no match		Unknown	
		IWB8638	Not Mapped	4.77E-05	1	0.02	0.07	no match		Unknown	
		IWB46787*	Not Mapped	4.29E-05	1	3.22	11.93	Traes_5AL_A26E70EFB.1	1	Unknown	S
		IWB48775	Not Mapped	4.29E-05	1	s.a.	s.a.	Traes_5AL_217A5FE69.2	1	Unknown	N
		IWB58986	Not Mapped	4.29E-05	1	s.a.	s.a.	Traes_5DL_939F0D4E2.1	1	Unknown	S
		IWA2098*	6B 1.28cM	6.39E-05	1	2.89	10.70	Traes_6BS_EA1DF4148.1	1	Enhanced disease resistance 2-like protein	N
		IWB55921*	5A 459.12cM	7.86E-05	1	1.61	5.96	Traes_5AL_6BA7849E7.1	1	Unknown	S
		IWA2558	5A 737.3cM	1.21E-04	1	0.00	0.00	Traes_5AL_19637DE03.1	1	AP-2 complex subunit alpha-2- like protein	N
		IWB5567	5B 221.73cM	1.66E-04	1	0.67	2.48	Traes_5BL_F48317F91.1	1	Probable flavin-containing monooxygenase 1	S
		IWB7364	5B 221.73cM	1.66E-04	1	s.a.	s.a.	no match		Unknown	
		IWB43461	5B 221.73cM	1.66E-04	1	s.a.	s.a.	Traes_5AL_EA74144D2	1	Probable flavin-containing monooxygenase 1	S
L-Arginine	0.737	IWB56221*	Not Mapped	1.17E-05	1	12.69	17.22	Traes_5BL_F367A99A7.1	1	Basic leucine zipper 25	3′ UTR
		IWB65729	Not Mapped	1.17E-05	1	s.a.	s.a.	X80068.1		Basic leucin zipper 1	3′ UTR
Pentose alcohol III	0.686	IWB242*	3B 265.41cM	2.11E-06	0.44	22.27	32.46	Traes_3B_E23F2C86F.1	12	Unknown	N
		IWB1705	3D 50.05cM	2.11E-06	0.44	s.a.	s.a.	Traes_3AS_0A005B552.1	1	Eukaryotic translation initiation factor 3A	S
		IWB25161	Not Mapped	2.11E-06	0.44	s.a.	s.a.	Traes_3DS_6774BF63A.2	1	ABC transporter G family member 7 protein	S
		IWB32797	Not Mapped	2.11E-06	0.44	s.a.	s.a.	Traes_3AS_EA87ED745.1	1	Peroxisomal membrane protein PEX14	
		IWB35825	3B 56.41cM	2.11E-06	0.44	s.a.	s.a.	no match		Unknown	
		IWB56509	6A 156.81cM	2.11E-06	0.44	s.a.	s.a.	Traes_3DS_C4E8AD8B4.2	1	Cellulose synthase catalytic subunit	S
		IWB65026	3B 56.41cM	2.20E-05	0.43	0.29	0.42	Traes_3AS_82679750F.2	1	E3 ubiquitin- protein ligase SINA-like protein 4	S
		IWB35763*	Not Mapped	6.41E-05	0.46	0.85	1.24	Traes_3AS_4E919E6081.3	1	Putative histidine- rich Ca2+-binding protein	S
		IWB18115*	3B 278.07cM	1.01E-04	0.39	1.94	2.83	Traes_3AS_47780354D.2	1	Transcriptional activator DEMETER	Intron
L-Tyrosine	0.364	IWB49741*	2Dx 269.06cM	5.15E-07	1	17.36	47.69	no match		Unknown	
Oligo II	0.329	IWB6807*	2A 506.66cM	8.33E-06	1	14.73	44.77	Traes_2DL_AADC2EA0F.1	1	ABC transporter E family member 2 protein	S

**Fig. 6.**
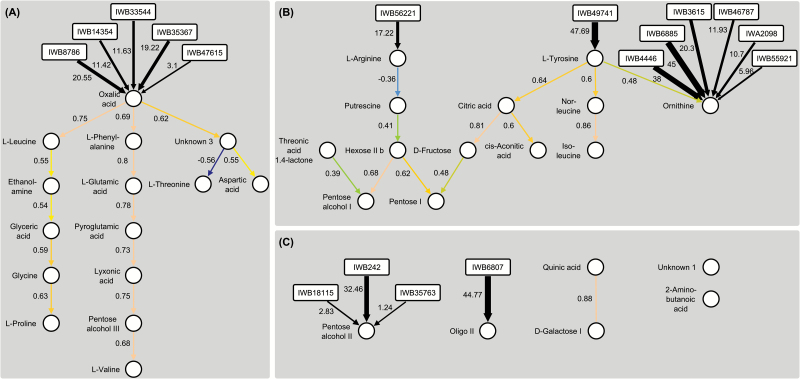
Partially directed network graphs describing the causal relationships among the 34 metabolites showing a significant genotypic variance (*P* < 0.05). The QPSO algorithm inferred two directed clusters (**A**) and (**B**) as well as some unrelated metabolites (**C**). SNP nodes are represented by rectangles and the proportion (%) of the explained genetic variance is both given as a number and indicated by the thickness of the connecting arrows. Metabolite nodes are represented by circles and correlations are displayed both numerically and graphically by coloured arrows (the colours correspond to the heat map in Fig. 3). High positive correlations are represented in earth tones and negative ones in blue.

Only mQTL associated with a *P* < 0.2 after FDR correction are listed. The chromosomal SNP positions refer to the reference annotations from the high-density 90k SNP array (Wang *et al.*, 2014). H^2^: heritability; R^2^: proportion (%) of phenotypic variance explained by the SNP; *p*_*G*_: proportion (%) of genetic variance explained by the SNP; Locus: GrainGenes locus according to chromosome maps available at http://wheat.pw.usda.gov/GG3/, last accessed 23 November 2016; Gene ID of candidate gene -search against ‘TriticumAestivum_IWGSC_map_AND_unmap_CDS.fa’ at http://pgsb.helmholtz-muenchen.de/plant/search.jsp last accessed 23 November 2016; Candidate gene product -source: http://plants.ensembl.org/, last accessed 23 November 2016; s.a.: similar as above, indicating highly linked SNPs; N: variation type non-synonymous; S: variation type synonymous; 3′ UTR: 3′ untranslated region; * significant SNPs (*P* < 0.1, FDR correction) used for the network analysis by the QPSO algorithm; please refer to Fig. 6.

The sequences in which the associated SNPs were embedded (Supplementary Table S4 at *JXB* online) were queried against available wheat genome sequence data to define a set of candidate genes, and against available plant gene and protein sequences (http://plants.ensembl.org/, last accessed 23 November 2016) to gather any known functional information. Only 21 of the sequences could be functionally assigned. The candidate genes encoded several transporters, catalytic enzymes, proteins involved in molecular signalling processes, transcriptional and translational regulators, and one defence-related protein. Most of the annotated genes are thought to generate just a single message, but one generates 12 splicing variants (Table 1). Additionally, we analysed the type of variation for the assigned SNPs. We detected a number of synonymous (S) and non-synonymous (N) variations as well as variation in the 3′ untranslated region (UTR) for two of the assigned SNPs and one SNP with an intron. The respective sites of variation and the corresponding amino acid changes are shown in Supplementary Table S4. We observed similar variation types for functionally similar SNPs, such as for a flavin-containing monooxygenase 1 (S) and for a basic leucine zipper (bZIP) protein (3′ UTR).

Ten mQTL were mapped for oxalic acid to chromosomes 5B, 7A, and 6A (Table 1). The strongest associations involved a chromosome 5B non-annotated sequence and an unmapped sequence encoding a GTP-binding protein. Two highly associated SNPs, co-locating to chromosome 7A, marked a gene encoding a transcription elongation factor (TFIIS). Weaker associations were noted for a chromosome 5B sequence, a non-annotated sequence harbouring four SNPs on chromosome 5B, and a chromosome 6A sequence encoding a galactose oxidase/kelch repeat protein.

In total, 17 mQTL were detected for ornithine (Table 1). The strongest associations related to six SNPs with a proportion of genotypic variance >38%, two of which resided in a pair of closely linked genes, both encoding a hexose carrier protein, located on chromosome 5A. Significant associations were also noted for sequences mapping to chromosome 5B and chromosome 6B; the latter has been annotated as encoding an enhanced disease resistance 2-like protein. Among the less strongly associated SNPs, three were unmapped but likely involved in Ca^2+^ transport, one lay in a gene of unknown function mapping to chromosome 5A, and three were in a single site on chromosome 5B which likely corresponds to a gene encoding a flavin-containing monooxygenase 1. Additional weakly associated sequences included one unmapped locus and a second mapping to chromosome 5A encoding an AP-2 complex subunit alpha-2-like protein, probably also involved in Ca^2+^ transport. Our results point to the importance of Ca^2+^ and sugar balance for ornithine metabolism.

Nine SNPs were significantly associated with variation for pentose alcohol III abundance. The strongest association involved a gene of unknown function mapping to chromosome 3B. A second locus mapping to chromosome 6A matched the site of a gene encoding a cellulose synthase catalytic subunit. Three other significant associations involved genes encoding for an ABC transporter G family member 7 protein, a peroxisomal membrane protein PEX14 (both unmapped), and the eukaryotic translation initiation factor 3A. Among the more weakly associated SNPs were one encoding for the transcription activator DEMETER, an E3 ubiquitin-protein ligase SINA-like protein 4, an unmatched SNP at the same chromosome position, and an unmapped SNP encoding for a putative calcium-binding protein. Overall, we concluded that the associations found for pentose alcohol III involved a strong transcriptional and translational regulation.

Two L-arginine mQTL were identified, the larger one explaining 17.2% of the variance. Both informative SNPs lay within genes encoding a bZIP protein, with both yet to be chromosomally mapped.

A high proportion of the variance was explained by the sequences associated with the L-tyrosine (47.69%) and oligo II (44.77%) mQTL. The oligo II mQTL was associated with a gene encoding an ABC-transporter protein.

### Network analysis revealed causal relationships among metabolites

Causal relationships were inferred from the reconstructed partially directed networks (Fig. 6). The five genes strongly associated with oxalic acid abundance appeared to exert a pleiotropic effect on a number of causally related metabolites, likely mediated by the concentration of oxalic acid (Fig. 6A). In the relevant metabolic network, oxalic acid lies directly upstream of L-leucine, L-phenylalanine, and an as yet unidentified metabolite (‘unknown 3’). The abundance of the latter directly affects the abundances of both L-threonine (negatively) and aspartic acid (positively). This causal relationship is of interest, because aspartic acid synthesis is thought to be catalysed by the reversible transamination of oxaloacetate, its direct precursor in the citric acid cycle; meanwhile, L-threonine is thought to be synthesized from aspartic acid via homoserine. On the other hand, oxalic acid is hydrolysed from oxaloacetate and not yet known to be a precursor or regulator of L-threonine and aspartic acid biosynthesis. In the second network branch, L-phenylalanine was shown to lie upstream of L-glutamic acid, pyroglutamic acid, lyxonic acid, pentose alcohol II, and L-valine. This constellation indicates control of various branches of amino acid biosynthesis by the concentration of L-phenylalanine, which in turn depends on that of oxalic acid. Ethanolamine, glyceric acid, glycine, and L-proline all lie downstream of L-leucine, indicating a relationship between the abundance of the branched amino acid L-leucine with that of the secondary amine L-proline, as well as with the abundance of certain glycolysis intermediates and derived small amino acids. Notably, the biosynthesis of both L-proline and L-leucine involves several enzymes acting on precursors of, respectively, L-glutamic acid and pyruvate. Because glycolysis uses precursors upstream of pyruvate, and pyruvate also lies upstream of L-glutamic acid, it seems unlikely that L-leucine exerts feedback control in both directions. Thus, the positioning of L-phenylalanine upstream of L-glutamic acid and L-valine seems to be more plausible. No functional test of these suggested causal relationships has been attempted so far, and the importance of oxalic acid in this network has yet to be confirmed.

In the second cluster (Fig. 6B), ornithine abundance was associated with 17 SNPs but only one causal relationship with the upstream L-tyrosine could be inferred. Ornithine is derived from L-glutamic acid, a side product of the citric acid cycle, while L-tyrosine is synthesized via the shikimate pathway, which lies upstream of the citric acid cycle; a proposed causal relationship between the abundance of ornithine and that of L-tyrosine therefore seems plausible. Similarly, the positioning of the isomers norleucine and isoleucine (derived from pyruvate) downstream of L-tyrosine, as well as that of citric acid, appears to be logical. A surprising link is the negative association established between L-arginine and its breakdown product putrescine with the hexose pool (hexose II and D-fructose), pentose I, and pentose alcohol I. This causal relationship was also linked to citric acid via D-fructose, prompting the suggestion that the gene associated with L-arginine abundance could influence amino acid catabolism via the ornithine pathway, as well as influencing the size of the pools of soluble carbon-related precursors of amino acid synthesis.

## Discussion

Continuing improvements in metabolomics and genomics have helped unravel the genetic basis of metabolic diversity in plants (Fiehn, 2002; Fernie and Schauer, 2009; Saito and Matsuda, 2010). While much of the effort has been focused on model species (Keurentjes *et al.*, 2006; Lisec *et al.*, 2009), progress has been recorded in maize (Wen *et al.*, 2014), rice (Luo, 2015), and barley (*Hordeum vulgare*) (Cockram *et al.*, 2010). Recent advances in developing the genomic toolbox in wheat (http://www.wheatgenome.org/, last accessed 23 November 2016) (Choulet *et al.*, 2014; Marcussen *et al.*, 2014; Pfeifer *et al.*, 2014) also paving the way for a deeper understanding of metabolic diversity in wheat (Hill *et al.*, 2015).

### Metabolite abundance is strongly influenced by genotype × environment interactions

The reproducibility of the metabolite profiling was in line with previous findings in maize (Riedelsheimer *et al.*, 2012a; Wen *et al.*, 2014). Riedelsheimer *et al.* (2012a) acquired GC-MS-based metabolite profiles for 130 compounds from leaves of 285 maize lines. Wen *et al.* (2014) included 184 annotated and 799 unknown metabolites detected on LC-MS from kernels of 702 maize genotypes. Thus, comparable precision can be reached independent of the plant species, organ, and the technology applied for metabolite profiling.

We observed pronounced environment × genotype interactions, which were expected because the metabolic status of a plant is governed by the concerted action of many enzymes, each of which can respond to the growing environment (Fiehn, 2002; Tohge and Fernie, 2010; Luo, 2015). Accordingly, significant variations in metabolite levels from durum wheat were attributed to genotype × environment interactions (Beleggia *et al.*, 2013). In tomato, the correlation with respect to metabolite abundance between the two seasons sampled was high for only about one half of the metabolites, a result revealing a substantial contribution of genotype × ripening stage interactions (Sauvage *et al.*, 2014). In contrast, a comprehensive analysis of rice metabolome diversity based on 840 metabolites collected for 529 accessions in two environments estimated a heritability of >0.5 for 59% of the metabolites and >0.7 for 24% of them (W Chen *et al.*, 2014). Similarly, when the performance of 175 rice lines was characterized at three environments, 68% of metabolite abundances were associated with a heritability of >0.5 (Matsuda *et al.*, 2015). The authors suggested that these high heritability values were mainly a result of the large diversity within the investigated set of rice genotypes.

### Metabolite correlations suggest a level of coordination between synthetic pathways

The strongest inter-metabolite correlations involved sugars, small organic acids, and amino acids (Fig. 3), a pattern seen also in Arabidopsis (Sulpice *et al.*, 2010), maize (Riedelsheimer *et al.*, 2012*b*; Wen *et al.*, 2015), tomato (Sauvage *et al.*, 2014), and rice (Matsuda *et al.*, 2012); which also relied on GC-MS technology for metabolite profiling. Because this approach favours the profiling of primary metabolites (Fiehn, 2002; Deborde *et al.*, 2011), the detection of the aforementioned compound classes is somehow predestined. However, besides this restriction to a sub-fraction of the metabolome, we could conclude that there exists coordinated control over sets of metabolites, representing key compounds, intermediates, or products of glycolysis and the citric acid cycle. Small organic acids related to the glycolate cycle and photorespiration behaved in a coordinated fashion, which implies a regulatory connection between photosynthesis and the citric acid/glycolate cycles. LC-MS-based metabolite profiling enables a larger structural diversity to be covered (Fernie and Schauer, 2009; Tohge and Fernie, 2010). This has made it possible to dissect the genetic components of polyamine and flavonoid biosynthesis in rice (Wen *et al.*, 2014) and to gain insights into the genetic architecture underlying natural variation in rice primary and secondary metabolism (W Chen *et al.*, 2014; Matsuda *et al.*, 2015). These advancements in speed and coverage will allow for more comprehensive and large-scale elucidation of metabolite and gene–metabolite correlations in the near future.

### GWAS can elucidate the genetic basis of metabolite abundance

Our GWAS revealed mQTL for six metabolites that did not exhibit significant interactions with the environment. None of them have formerly been reported in wheat (Hill *et al.*, 2015). The mQTL accounted for around 30% of the variance for ornithine, L-tyrosine, and oligo II, and for over 58% of that for oxalic acid, L-arginine, and pentose alcohol III (Supplementary Table S2). Similar results have been reported in barley (Cockram *et al.*, 2010). Assuming a high degree of genetic complexity for the metabolic traits in our data set, we conclude that only a small proportion of the SNPs that control polygenic traits were likely to be detected using our approach.

The 40 informative SNPs were dispersed unevenly over the genome (Table 1), and there was not a single case of an individual SNP being associated with more than one metabolite, unlike what has been observed in cognate studies on primary and secondary metabolites (Bergelson and Roux, 2010; Matsuda *et al.*, 2012; Sauvage *et al.*, 2014). Most of the metabolites were found to be tightly associated with only a small number of strong mQTL in our study, which might hint at mechanisms which confer tight control over these metabolite levels.

With respect to the four sequences associated with oxalic acid abundance, one putatively encodes TFIIS (a transcription elongation factor), one a GTP-binding protein, and one a galactose oxidase/kelch repeat protein. TFIIS enhances the activity of RNA polymerase II by reactivating transcription elongation complexes in which transcription has been arrested (Booth *et al.*, 2000). In Arabidopsis TFIIS loss-of-function mutants, only around 2.3% of genes have altered transcription. This includes the upregulation of genes involved in glycoside, glucosinolate, and general metabolism and the downregulation of genes controlling secondary metabolism and the synthesis of amino acids and aromatic compounds (Grasser *et al.*, 2009). It is unclear in which direction TFIIS affects oxalic acid levels. GTP-binding proteins are signalling proteins involved in the control of cell division, cell cycling, ribosome assembly, and protein synthesis (Miyawaki and Yang, 2014; Urano and Jones, 2014). In the filamentous fungi *Botrytis cinerea*, mutants lacking the expression of a small GTPase (BcCdc42) had impaired nuclear division, germination, and virulence, and a reduced ability to secrete oxalic acid (Kokkelink *et al.*, 2011). Aluminium (Al) tolerance in wheat is highly correlated with an Al-activated release of small organic acids, such as citric acid, malic acid, and oxalic acid, and oxalic acid is also involved in intracellular Al detoxification. Genetic markers linked to Al tolerance loci are shared between rice, wheat, and barley (Kochian *et al.*, 2004). A possible involvement of GTPase signalling in the Al ion-induced secretion of organic acids in both Arabidopsis and rye (*Secale cereale*) has only recently been

 reported (YY Li *et al.*, 2015). Thus the GTPase gene highlighted by our GWAS could function in Al-mediated oxalic acid level induction. Finally, galactose oxidase/kelch repeat proteins could be connected to oxalic acid abundance through their oxidation of primary alcohols, although no *a priori* experimental evidence for this has been found in the literature.

With respect to ornithine abundance, the candidate sequences identified encoded two hexose carrier proteins, closely linked to one another on chromosome 5A. These proteins belong to a large family of membrane proteins responsible for the binding and transport of various carbohydrates, organic alcohols, and acids (Silverman, 1991). The strong association between these loci and ornithine abundance implies that one or both gene products are intimately involved in the regulation of ornithine level. Two of the three informative SNPs lay within a chromosome 5B sequence encoding flavin-containing monooxygenase 1. Flavin monooxygenases catalyse a wide range of oxygenation reactions and have been classified into eight groups (Huijbers *et al.*, 2014), one of which includes the YUCCAs (EC 1.14.13.168), which closely resemble ornithine monooxygenases at the primary sequence level. All plants contain these enzymes (Dai *et al.*, 2013).

The two genes associated with L-arginine abundance both encoded bZIP proteins. Members of the bZIP superfamily of eukaryotic DNA-binding transcription factors act as master regulators of a large number of developmental and physiological processes (Alves *et al.*, 2013). The wheat genome harbours at least 187 bZIP genes (X Li *et al.*, 2015), one of which (*TabZIB113*) is identical to Traes_5BL_F367A99A7.1. This gene was differentially transcribed during wheat anther development, but its function is not known.

Genes associated with the abundance of pentose alcohol III were distributed across the genome. The putative candidate genes included one encoding a cellulose synthase catalytic subunit, a eukaryotic translation initiation factor 3A, a DEMETER transcription activator, an E3 ubiquitin-protein ligase SINA-like protein 4, and a putative calcium-binding protein. Further potentially informative associations involved two unmapped genes, one encoding a member of the G family of ABC transporters and the other the peroxisomal membrane protein PEX14. Similarly, a gene encoding a member of the E family of ABC transporters was identified as a possible candidate for an oligo II mQTL. The implied comparable regulatory mechanisms for these two sugars is reasonable, given the importance of sugars for growth (Eveland and Jackson, 2012; Kadereit *et al.*, 2014). Nevertheless, as yet it cannot be determined whether the products of any of these genes act as direct mediators of sugar level or whether they form part of the relevant regulatory machinery.

### Combining genotypic and phenotypic data uncovers novel causal relationships

While conventional correlation analysis effectively identifies interactions between metabolites or between metabolites and enzymes or genes (undirected), it often neglects the underlying metabolic and genetic networks (causalities, directed) (Sulpice *et al.*, 2010; Tohge and Fernie, 2010; Wang and van Eeuwijk, 2014). Using recent methodology (Wang and van Eeuwijk, 2014), we revealed genetic interventions on groups of metabolites in wheat. Our analysis, based on a set of statistically significant mQTL (*P* < 0.1 corrected for FDR), identified oxalic acid as a key regulator or precursor of various branches of amino acid synthesis. Oxalic acid is hydrolysed via three pathways that involve glyoxylate/glycolic acid, ascorbic acid, and oxaloacetate (Kadereit *et al.*, 2014), all of which are derived from the citric acid cycle. From the prominent position of oxalic acid in our partially directed network, we conclude that oxalic acid exerts feedback control over a number of steps in amino acid biosynthesis. Experimental evidence was provided by an *in vitro* study showing that oxalic acid improves protein synthesis by enhancing the ATP supply in a cell-free system derived from *Escherichia coli* (Kim and Swartz, 2000). Further support is supplied by the known activity of oxalic acid as a signalling molecule for a number of host–pathogen interactions (Lehner *et al.*, 2008), as well as from its direct involvement in conferring tolerance to abiotic stress (Kochian *et al.*, 2004). In Arabidopsis, Sulpice *et al.* (2010) have documented strong correlations between the synthesis of amino acids and the abundance of malic acid and some other small organic acids – but, notably, not oxalic acid.

The negative relationship between the abundance of L-arginine and its breakdown product putrescine with a side-link to upstream L-tyrosine (Fig. 6) has not been reported for any other plant species to date. Toubiana *et al.* (2012) observed a negative correlation between the putrescine and maltose content in tomato, while indirect evidence for the regulatory activity exerted by putrescine over several synthetic pathways comes from significant epistatic effects associated with its abundance in maize (Wen *et al.*, 2015).

Overall, the outcome supports an extensive co-regulation of central pathways in both N and C metabolism, as previously postulated (Gutiérrez *et al.*, 2007; Toubiana *et al.*, 2012). However, experimental evidence on the detected SNP-metabolite relationships is needed to confirm the postulated regulatory networks. Ideally, such studies include the analysis of mutant or introgression lines, as has been done in pioneering works for Arabidopsis (Keurentjes *et al.*, 2006; Tohge and Fernie, 2010) and rice (Gong *et al.*, 2013; W Chen *et al.*, 2014), as well as re-sequencing approaches as reported for maize (Wen *et al.*, 2014). Similar prospects can be anticipated for wheat in the near future owing to the advancements in cereal transformation and sequencing technologies.

## Conclusions

A number of new gene–metabolite associations and their causal relationships have been revealed in this study, confirming the potential of the metabolite GWAS approach even in a polyploid species. The limitations of this approach relate in part to the complex inheritance of metabolite abundance and in part to the limited availability of sets of genetic markers and analysable metabolites. These constraints could be overcome by additional genomic marker development and fine-mapping based on new deep-sequencing and transcriptomics technologies (Díaz *et al.*, 2012; Kahl *et al.*, 2012; Würschum *et al.*, 2015). Technical improvements to metabolomic platforms (Saito and Matsuda, 2010; Deborde *et al.*, 2011; Tohge *et al.*, 2015) should be able to address the latter restriction, and some progress in this direction has been made in both maize (Wen *et al.*, 2014) and rice (Matsuda *et al.*, 2015). More sophisticated choices of germplasm can enhance the effectiveness of GWA approaches, as demonstrated in rice, for which a standard association panel has been combined with a number of conventional mapping populations (W Chen *et al.*, 2014; Huang *et al.*, 2010). In turn, these sophisticated functional genomics approaches rely on the establishment of novel high-throughput phenotyping platforms (D Chen *et al.*, 2014). The recent extension of marker-assisted selection by powerful genomic selection approaches has proven applicable to metabolite markers, although with reduced accuracy when compared to genomic markers. The expectation is for further advances in knowledge-based breeding strategies, predicated on the idea of predicting plant performance from combinations of markers, which will further facilitate crop improvement in the years to come.

## Author contributions

AM, H-PM, JCR, EE, VK, FL, RS, EK, and JS designed the research; AM, GL, YJ, YZ, and AH performed the research and analysed data; EE, RS, EK, and JS performed field trials and provided plant material; AM, GL, and JCR wrote the paper; HW provided support for the network analysis. The authors declare no conflict of interest.

## Supplementary Material

Supplementary Data
