# Supplementary material for: Genome–metabolite associations revealed low heritability, high genetic complexity, and causal relations for leaf metabolites in winter wheat (Triticum aestivum)
Source: J Exp Bot. 2016 Dec 22;68(3):415–28. doi: 10.1093/jxb/erw441 (PMC5441906; doi:10.1093/jxb/erw441)
Supplement: Supplementary Data [file erw441_Supplementary_Data.zip › supplementary_figures_S1_S7_tables_S1_S4.pdf]

Supplemental Data for Research Paper

Genome-metabolite associations revealed low heritability, high genetic complexity, and causal relations for leaf metabolites in winter wheat

Andrea Matros[a], Guozheng Liu[b], Anja Hartmann[c], Yong Jiang[b], Yusheng Zhao[b], Huange Wang[d], Erhard Ebmeyer[e], Viktor Korzun[e], Ralf Schachschneider[f], Ebrahim Kazman[g], Johannes Schacht[h], Friedrich Longin[i] Jochen Christoph Reif[b], Hans-Peter Mock[a,1],

[a] Department of Physiology and Cell Biology, Applied Biochemistry, Leibniz Institute of Plant Genetics and Crop Plant Research, 06466 Gatersleben, Germany

[b] Dept. Breeding Research, Quantitative Genetics, Leibniz Institute of Plant Genetics and Crop Plant Research, 06466 Gatersleben, Germany

[c] Department of Physiology and Cell Biology, Molecular Plant Nutrition, Leibniz Institute of Plant Genetics and Crop Plant Research, 06466 Gatersleben, Germany

[d] Biometris, Department of Plant Sciences, Wageningen University, 6708 PB Wageningen, The Netherlands

[e] KWS LOCHOW GMBH, 29296 Bergen, Germany

[f] Nordsaat Saatzuchtgesellschaft mbH, 38895 Langenstein, Germany

[g] Lantmännen SW Seed Hadmersleben GmbH, 39398 Hadmersleben, Germany

[h] Limagrain GmbH, 31226 Peine-Rosenthal, Germany

[i] University of Hohenheim, State Plant Breeding Institute, 70599 Stuttgart, Germany

matros@ipk-gatersleben.de, liug@ipk-gatersleben.de, hartmann@ipk-gatersleben.de, jiang@ipk-gatersleben.de, zhao@ipk-gatersleben.de, hw428@cam.ac.uk, ebmeyer@kws-lochow.de, viktor.korzun@kdws.de, r.schachschneider@nordsaat.de, ebrahim.kazman@syngenta.com, johannes.schacht@limagrain.de, Friedrich.Longin@uni-hohenheim.de, reif@ipk-gatersleben.de, mock@ipk-gatersleben.de

[1]Corresponding author:

PD Dr. Hans-Peter Mock

IPK-Gatersleben, Dept. Physiology and Cell Biology, Applied Biochemistry

Corrensstr. 3, D-06466 Gatersleben

Phone: +49 39482 5506

E-mail: mock@ipk-gatersleben.de

Supplemental Figure 1: Distribution of the Box–Cox transformed levels of the 76 individual metabolites across the three locations. Identity of the metabolites is given in Supplemental Table 2. Red frames indicate metabolites for which significant associations have been detected.

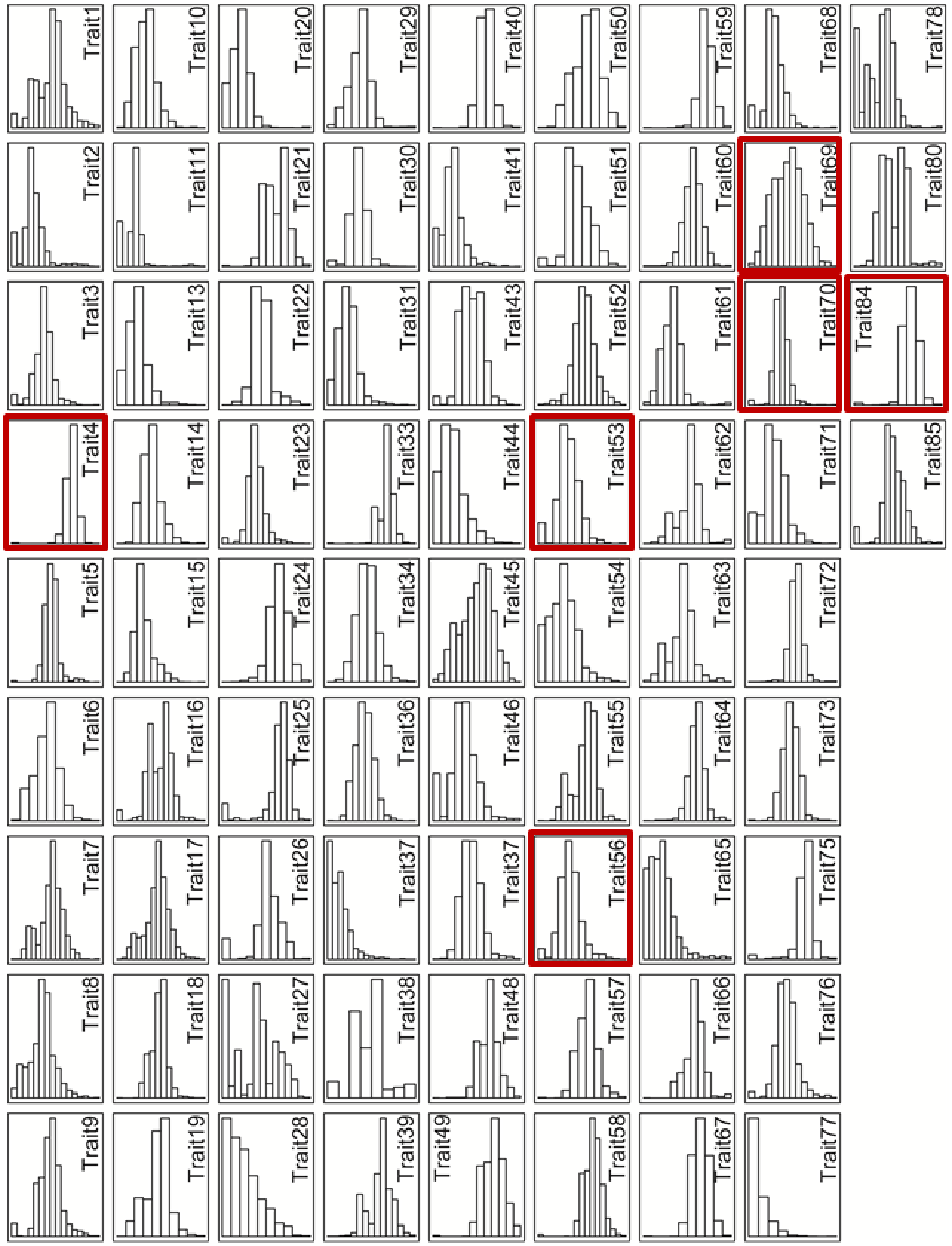

Supplemental Figure 2: Representative GC-MS chromatogram of wheat flag leaf.

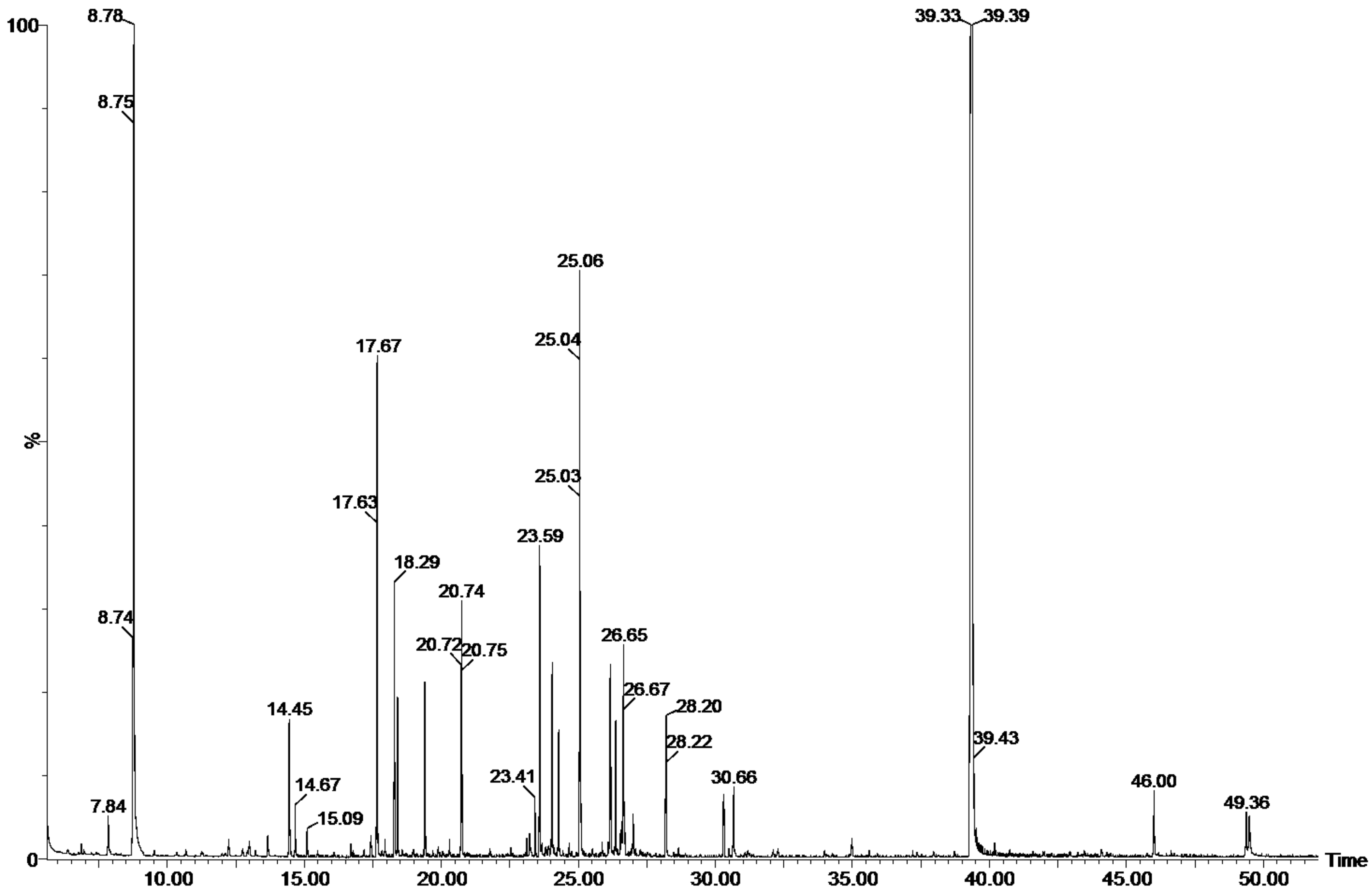

Supplemental Figure 3: (A) Metabolite correlations with principle component (PC) 1 and PC2. (B) Loadings plot of the PCA.

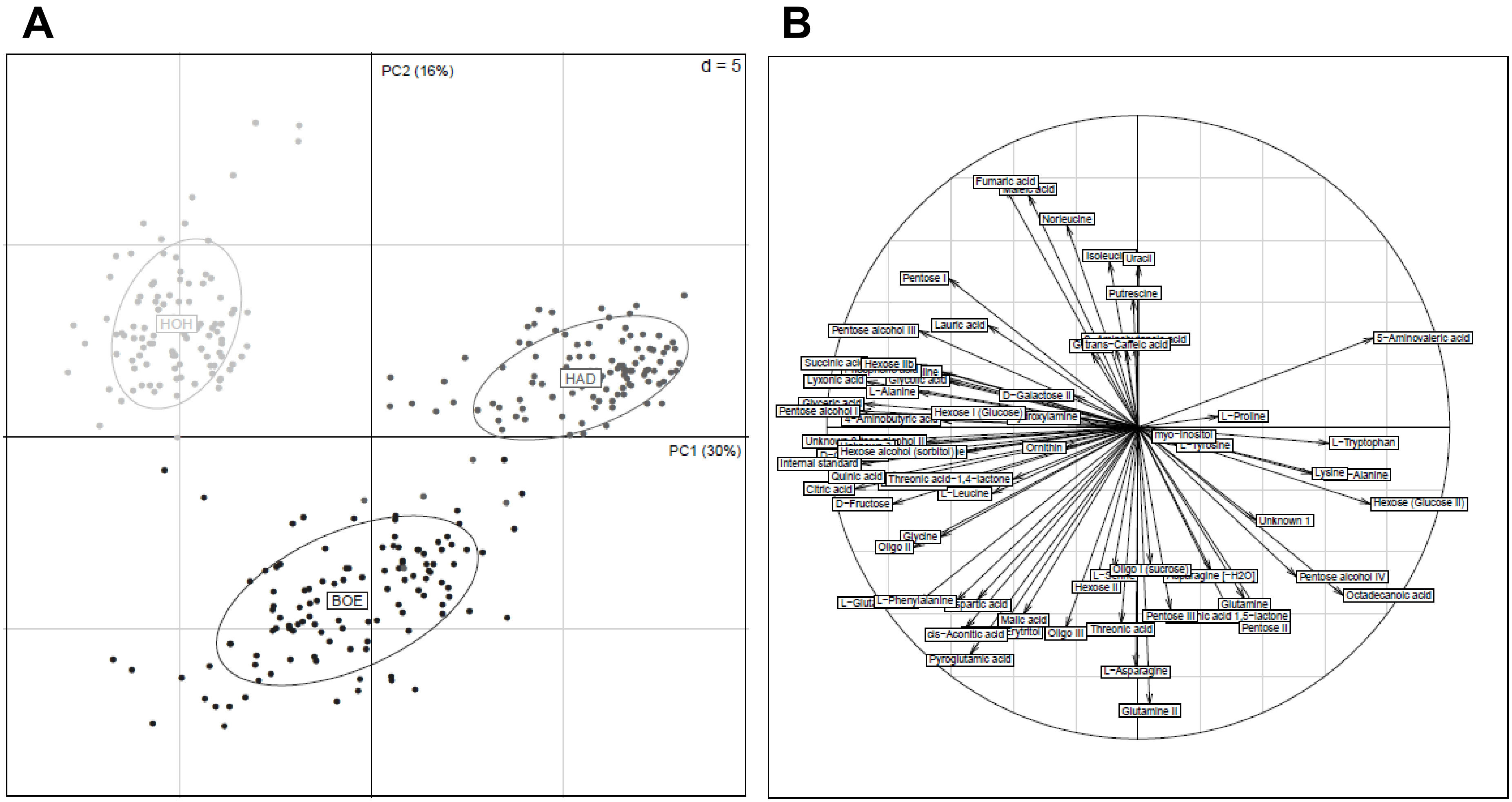

Supplemental Figure 4: Individual metabolite profiles across the 135 investigated lines for the three locations. A blue frame indicates significance levels of genotypic variance < 0.05, while an orange frame indicates very low heritability. Please also refer to statistical data provided in Supplemental Table 2.

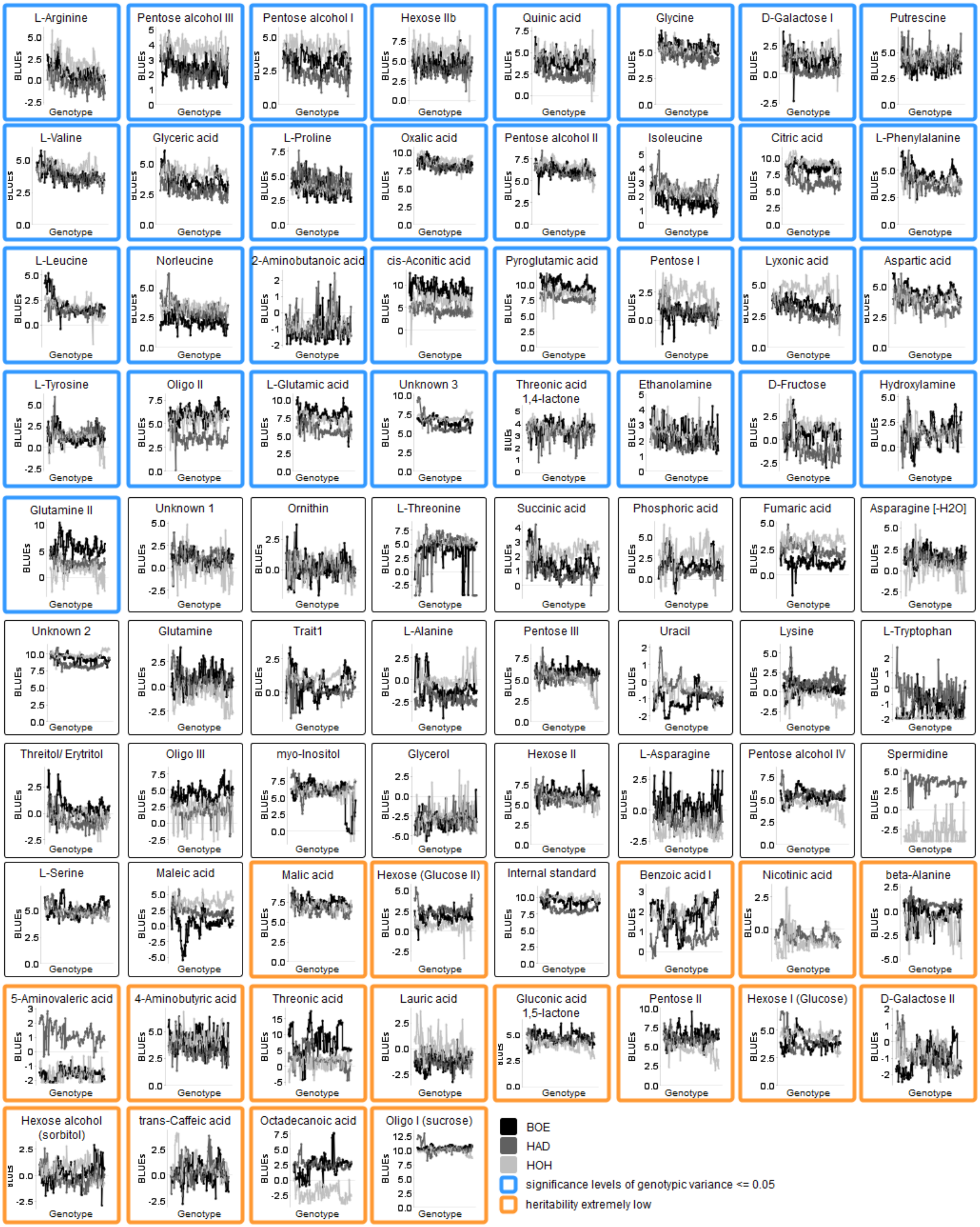

Supplemental Figure 5: Heritability of metabolites separated for groups of lines with different heading date across all locations. The 135 winter wheat lines were assembled into three groups of heading time (early, middle and late). The mean values, with 0.29 for early, 0.23 for middle and 0.21 for late heading time are indicated as dotted lines.

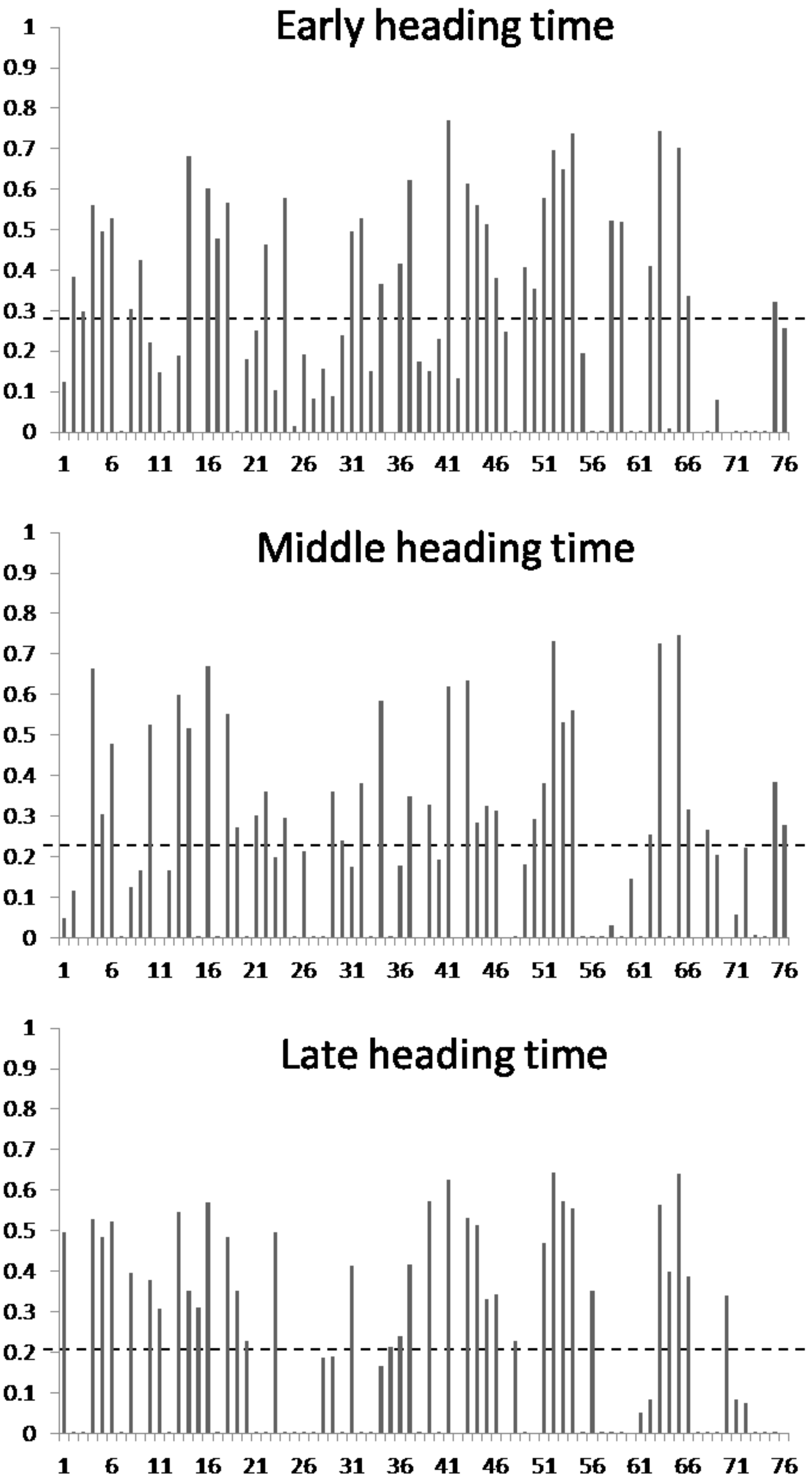

Supplemental Figure 6: Annotated correlation-based hierarchical cluster analysis corresponding to the pairwise Pearson correlation pattern presented in Fig. 3.

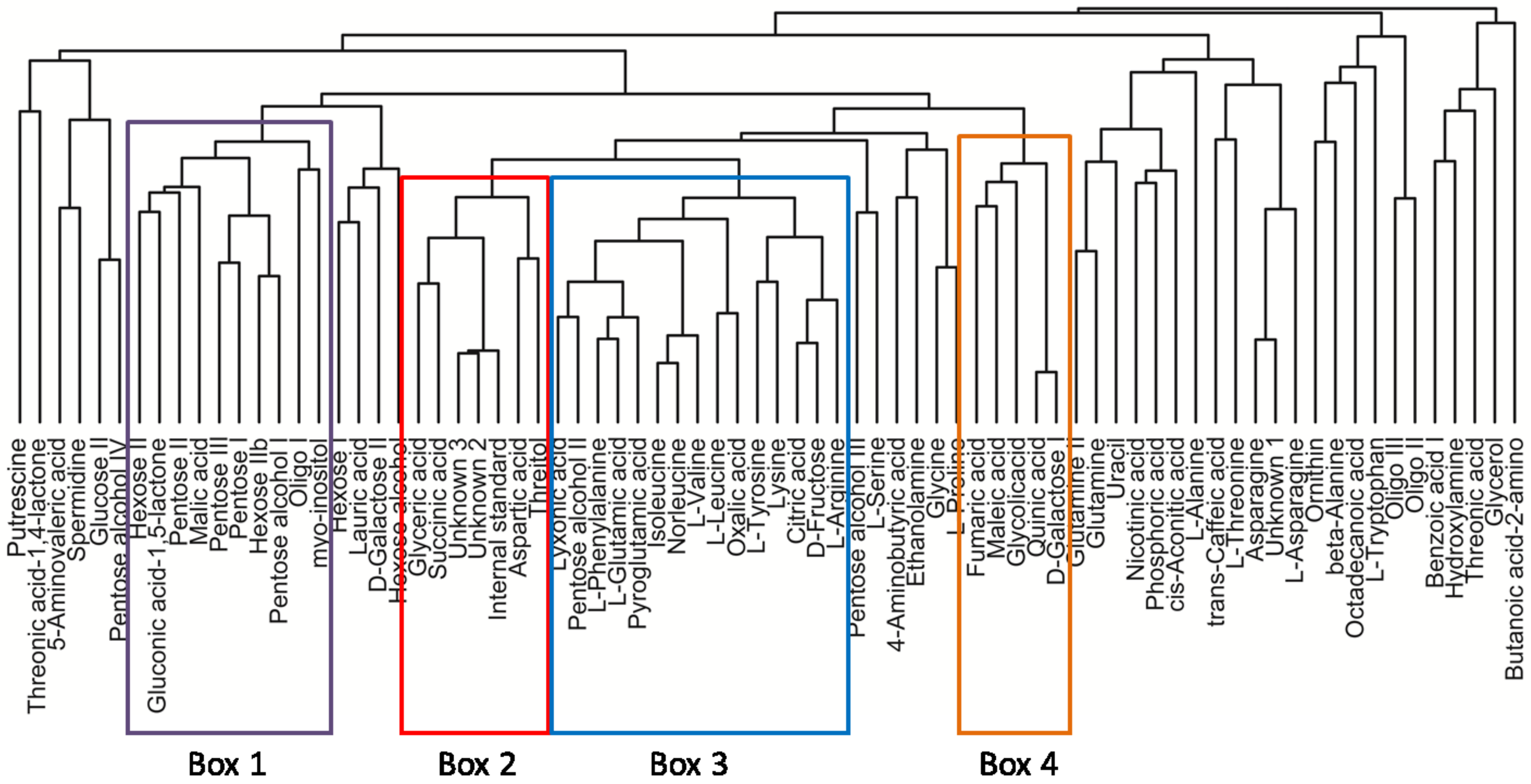

Supplemental Figure 7: Associations among the 135 wheat inbred lines revealed by complete linkage clustering method based on Euclidean distances estimated based on metabolite abundance (A) and Rogers' distances estimated based on the genomic data (B).

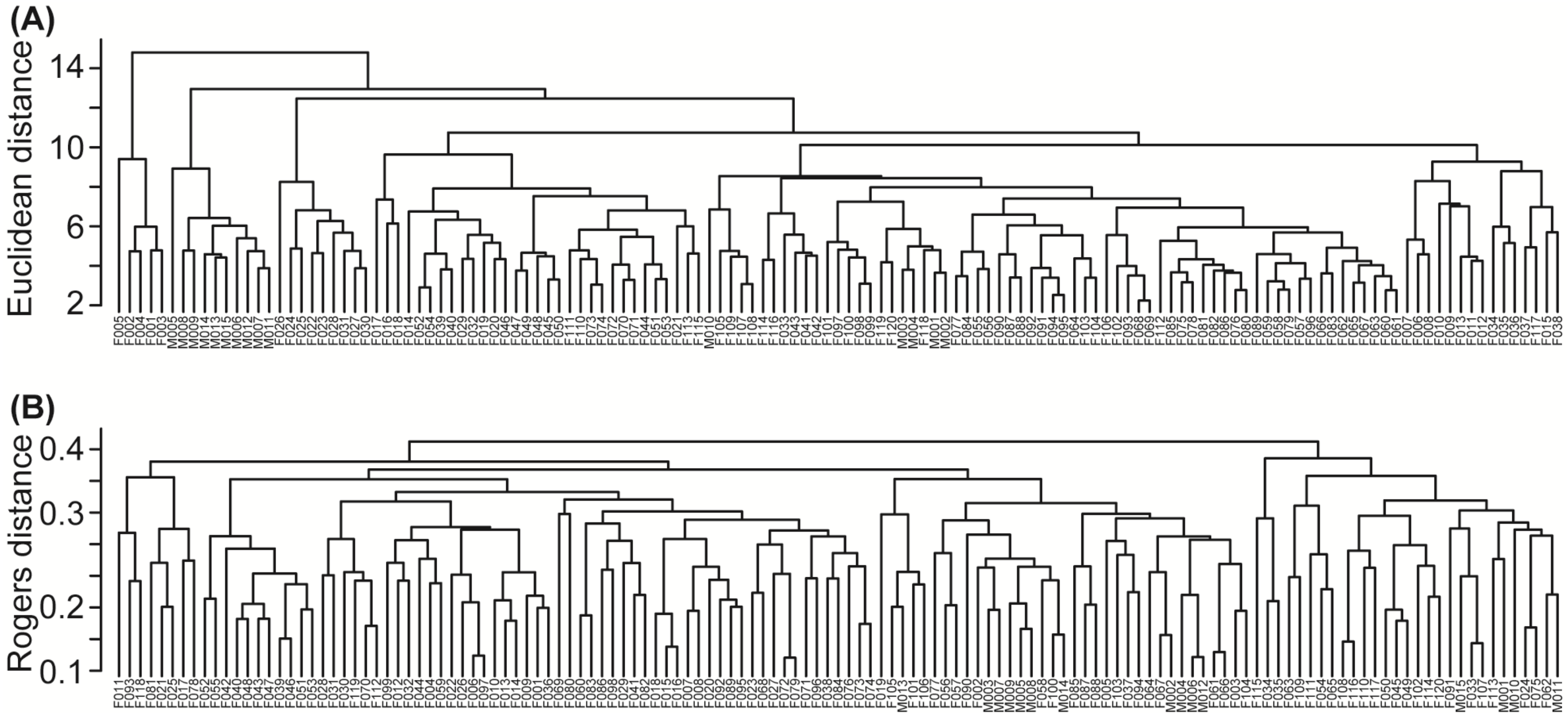

Supplemental Table 1: List of measured metabolites. The trait #, compound name, molecular mass of the quantified fragment ion (*m/z*) and the retention index (RI) are listed for the 76 metabolites analyzed in this study. The abbreviations Oligo I, Oligo II and Oligo III relate to sugar oligomers with increasing degree of polymerization. RI of Oligo III was not determined (nd) as calibration mix range was exceeded Rows marked with blue show traits whose significance levels of genotypic variance are lower than 0.05. Rows marked with red show traits whose heritability are extremely low.

Trait #	compound name	TMS	Molecular fragment mass [*m/z*]	Retention Index (RI)
Trait1	Glycolic acid	2	205.1	1079
Trait2	L-Alanine	2	116.0	1100
Trait3	Hydroxylamine	3	249.1	1112
Trait4	Oxalic acid	1	220.1	1138
Trait5	L-Leucine	2	158.0	1138
Trait6	L-Valine	2	144.1	1213
Trait7	Benzoic acid I	1	179.1	1243
Trait8	Ethanolamine	3	174.1	1264
Trait9	Phosphoric acid	3	314.1	1268
Trait10	Norleucine	2	158.1	1269
Trait11	Glycerol	3	205.0	1273
Trait13	Nicotinic acid	1	180.1	1290
Trait14	Isoleucine	2	158.1	1290
Trait15	L-Proline	2	142.1	1293
Trait16	Maleic acid	2	245.1	1300
Trait17	Glycine	3	174.1	1302
Trait18	Succinic acid	2	247.1	1311
Trait19	Glyceric acid	3	189.1	1328
Trait20	Uracil	2	241.1	1332
Trait21	Fumaric acid	2	245.1	1348
Trait22	L-Serine	3	204.1	1359
Trait23	Unknown 1		141.1	1366
Trait24	Threonic acid-1,4-lactone	2	247.1	1368
Trait25	L-Threonine	3	218.0	1385
Trait26	beta-Alanine	3	174.1	1426
Trait27	Spermidine	5	143.1	1449
Trait28	5-Aminovaleric acid	3	174.1	1455
Trait29	Malic acid	3	233.1	1488
Trait30	Asparagine [-H2O]	2	115.1	1496
Trait31	Threitol/ Erytritol	4	217.1	1510
Trait33	Pyroglutamic acid	2	156.1	1514
Trait34	Aspartic acid	3	218.1	1519
Trait36	4-Aminobutyric acid	3	174.1	1527
Trait37	Butanoic acid, 2-amino-	2	130.0	1527
Trait38	Threonic acid	4	292.0	1562
Trait39	L-Glutamic acid	3	246.1	1619
Trait40	L-Phenylalanine	2	218.1	1621
Trait41	Lauric acid	1	204.1	1650

Trait43	Pentose I	4	103.1	1665
Trait44	L-Asparagine	3	116.1	1667
Trait45	Pentose alcohol I	5	217.1	1727
Trait46	Glutamine	3	155.1	1733
Trait47	Putrescine	4	174.1	1735
Trait48	Pentose alcohol II	6	147.1	1741
Trait49	cis-Aconitic_acid	3	73.0	1749
Trait50	Lyxonic acid	5	103.1	1764
Trait51	Glutamine II	3	155.1	1770
Trait52	Gluconic acid 1,5-lactone	4	217.1	1782
Trait53	Ornithine	4	142.1	1816
Trait54	D-Fructose	5	437.2	1818
Trait55	Citric acid	4	273.1	1818
Trait56	L-Arginine	4	157.0	1821
Trait57	D-Galactose I	5	191.1	1857
Trait58	Quinic acid	5	345.2	1859
Trait59	Pentose II	5	103.1	1873
Trait60	Pentose III	5	103.1	1883
Trait61	Hexose I (Glucose)	5	204.1	1891
Trait62	Unknown 2		210.1	1894
Trait63	Internal standard		327.2	1895
Trait64	Hexose II	5	319.2	1898
Trait65	D-Galactose II	5	191.1	1907
Trait66	Unknown 3		210.1	1912
Trait67	Hexose II_b	5	319.2	1915
Trait68	Lysine	4	174.1	1917
Trait69	Pentose alcohol III	6	205.1	1930
Trait70	L-Tyrosine	3	218.1	1937
Trait71	Hexose alcohol (sorbitol)	1	319.2	1938
Trait72	Pentose alcohol IV	5	103.1	1977
Trait73	Hexose (Glucose II)	5	204.0	1977
Trait75	myo-inositol	6	305.1	2089
Trait76	trans-caffeic acid	3	219.0	2133
Trait77	L-Tryptophan	3	202.1	2204
Trait78	Octadecanoic acid	1	341.3	2241
Trait80	Oligo I (sucrose)	8	361.0	2639
Trait84	Oligo II	6	204.1	3142
Trait85	Oligo III	6	204.1	nd

Supplemental Table 2: Second degree statistics of the metabolite. The repeatability for the three individual locations, the environmental variance, the genotypic variance, the significance level of the genotypic variance, the genotype by environment interaction variance, the significance level of the genotype by environment interaction variance, the variance of the residuals and the heritability are listed for the 76 investigated metabolites. Rows marked in blue show traits whose significance levels of genotypic variance are lower than 0.05, and rows marked in red show traits whose heritability is extremely low.

Compound name	Trait #	Repeatability Hadmersleben	Repeatability Böhnshausen	Repeatability Hohenheim	Environmental Variance	Genotypic Variance	Significance of Genotypic Variance	Genotype x Environment Interaction Variance	Significance of Genotype x Environment Interaction Variance	Variance of Residuals	Heritability
L-Arginine	Trait56	0.7856	0.8005	0.7760	0.9454	0.4875	0.0000	0.2464	0.0000	1.0243	0.7373
Pentose alcohol III	Trait69	0.5812	0.8219	0.7246	0.4929	0.1843	0.0000	0.1314	0.0000	0.4496	0.6865
Pentose alcohol I	Trait45	0.5112	0.7977	0.7708	0.6923	0.1345	0.0000	0.1114	0.0000	0.3876	0.6516
Hexose II_b	Trait67	0.5947	0.7659	0.7689	0.8661	0.4168	0.0000	0.3827	0.0000	1.1779	0.6409
Quinic acid	Trait58	0.4363	0.7478	0.7165	0.7938	0.2545	0.0000	0.1964	0.0000	1.0780	0.6099
Glycine	Trait17	0.6834	0.6746	0.4226	0.0000	0.0874	0.0000	0.0581	0.0000	0.4135	0.6067
Putrescine	Trait47	0.7568	0.7452	0.6840	0.0830	0.1641	0.0000	0.1888	0.0000	0.5665	0.5905
D-Galactose I	Trait57	0.5641	0.7582	0.5901	0.0668	0.1378	0.0000	0.1389	0.0000	0.5929	0.5804
Oxalic acid	Trait04	0.3870	0.6402	0.5372	0.0269	0.1099	0.0000	0.0211	0.3074	0.7838	0.5788
Pentose alcohol II	Trait48	0.4315	0.5642	0.6486	0.0633	0.1086	0.0000	0.0935	0.0000	0.6385	0.5496
L-Proline	Trait15	0.8719	0.7950	0.6417	0.0000	0.1596	0.0000	0.2737	0.0000	0.4493	0.5481
L-Valine	Trait06	0.7547	0.6744	0.5965	0.0763	0.0795	0.0000	0.1059	0.0000	0.3619	0.5394
Citric acid	Trait55	0.5709	0.7597	0.6392	1.0453	0.1680	0.0000	0.2353	0.0000	0.8334	0.5229
Glyceric acid	Trait19	0.7005	0.7577	0.4858	0.1733	0.0813	0.0000	0.1141	0.0000	0.4224	0.5167
Isoleucine	Trait14	0.7235	0.8280	0.5359	0.1637	0.0665	0.0000	0.1229	0.0000	0.3000	0.4943
L-Phenylalanine	Trait40	0.6350	0.5674	0.6356	0.0399	0.1065	0.0000	0.1171	0.0000	0.7988	0.4883
Norleucine	Trait10	0.5800	0.8183	0.5756	0.4299	0.0607	0.0000	0.1318	0.0000	0.3704	0.4402
L-Leucine	Trait05	0.5453	0.5046	0.7377	0.0000	0.1072	0.0000	0.1710	0.0000	0.8828	0.4382
Pentose I	Trait43	0.5848	0.7157	0.6445	0.8848	0.1162	0.0001	0.2329	0.0000	0.9057	0.4223
Butanoic acid. 2-amino-	Trait37	0.5556	0.4781	0.5461	0.0396	0.0843	0.0001	0.1317	0.0000	0.8353	0.4149
Pyroglutamic acid	Trait33	0.5868	0.5902	0.7851	0.6744	0.1469	0.0002	0.2639	0.0000	1.2032	0.4262

cis-Aconitic_acid	Trait49	0.7210	0.7720	0.8030	5.1013	0.3431	0.0002	0.9635	0.0000	1.7913	0.4157
Lyxonic acid	Trait50	0.4615	0.6599	0.7117	0.4429	0.0691	0.0004	0.1567	0.0000	0.5419	0.4062
Aspartic acid	Trait34	0.3711	0.5657	0.5231	0.1011	0.0755	0.0005	0.1150	0.0000	0.8248	0.4008
L-Tyrosine	Trait70	0.4696	0.7232	0.7242	0.0000	0.1133	0.0017	0.3183	0.0000	1.0065	0.3645
L-Glutamic acid	Trait39	0.5656	0.6001	0.5671	0.3723	0.1061	0.0058	0.2967	0.0000	1.2817	0.3313
Oligo II	Trait84	0.4354	0.7230	0.7046	1.5645	0.0767	0.0066	0.2379	0.0000	0.8403	0.3293
Unknown 1	Trait23	0.6008	0.5106	0.7198	0.3500	0.1005	0.0114	0.3041	0.0000	1.4100	0.3055
Threonic acid-1.4-lactone	Trait24	0.2868	0.6821	0.5329	0.0000	0.0481	0.0118	0.1257	0.0000	0.6734	0.3182
Unknown 3	Trait66	0.3372	0.9009	0.4249	0.0168	0.0454	0.0220	0.2316	0.0000	0.4457	0.2788
D-Fructose	Trait54	0.6706	0.6367	0.5815	1.2767	0.1362	0.0263	0.5872	0.0000	1.8153	0.2756
Ethanolamine	Trait08	0.7173	0.6852	0.6257	0.0000	0.0385	0.0287	0.1805	0.0000	0.4556	0.2757
Ornithin	Trait53	0.3526	0.5520	0.6328	0.0000	0.0748	0.0330	0.2492	0.0000	1.3730	0.2661
L-Threonine	Trait25	0.7281	0.9538	0.7437	0.3876	0.6153	0.0467	4.7219	0.0000	1.4776	0.2579
L-Tryptophan	Trait77	0.7306	0.6827	0.3745	0.4233	0.0117	0.0518	0.0169	0.0529	0.4929	0.1693
L-Serine	Trait22	0.4576	0.4872	0.5674	0.0000	0.0254	0.0658	0.0809	0.0000	0.5961	0.2395
Asparagine [-H2O]	Trait30	0.5982	0.6992	0.7401	0.4834	0.0765	0.0778	0.4353	0.0000	1.5363	0.2127
Phosphoric acid	Trait09	0.4962	0.6263	0.6878	0.6308	0.0918	0.1010	0.5145	0.0000	2.2797	0.1964
Glutamine II	Trait51	0.7000	0.6476	0.5811	5.0341	0.1133	0.1646	0.8846	0.0000	2.8717	0.1705
Unknown 2	Trait62	0.2067	0.3839	0.7143	0.0679	0.0172	0.1745	0.0794	0.0000	0.6792	0.1634
Glycolic acid	Trait01	0.4410	0.3345	0.4674	0.0669	0.0244	0.1846	0.0888	0.0001	1.0658	0.1626
Pentose III	Trait60	0.2007	0.6061	0.8121	0.2773	0.0387	0.2788	0.3847	0.0000	0.9583	0.1526
Succinic acid	Trait18	0.6607	0.5842	0.3247	0.3197	0.0160	0.2942	0.1284	0.0000	0.6806	0.1332
L-Alanine	Trait02	0.5558	0.3959	0.3158	0.7039	0.0451	0.3299	0.2762	0.0000	2.4395	0.1265
L-Asparagine	Trait44	0.4420	0.5978	0.5148	0.8178	0.0261	0.3546	0.2160	0.0000	1.2112	0.1263
Uracil	Trait20	0.5178	0.6468	0.6230	0.1185	0.0113	0.3701	0.1289	0.0000	0.3992	0.1252
Glutamine	Trait46	0.4433	0.5309	0.6843	0.8430	0.0435	0.4038	0.5222	0.0000	1.7001	0.1176
Fumaric acid	Trait21	0.5418	0.6921	0.5074	1.2351	0.0119	0.4176	0.1457	0.0000	0.4871	0.1140
Oligo III	Trait85	0.2915	0.6853	0.6576	1.9609	0.0606	0.4218	0.7468	0.0000	2.8075	0.1079
Lysine	Trait68	0.4466	0.5836	0.6733	0.6599	0.0275	0.4257	0.3534	0.0000	1.0684	0.1143
myo-inositol	Trait75	0.5322	0.3341	0.7171	0.0000	0.0751	0.4458	0.6538	0.0000	4.0760	0.1129

Hydroxylamine	Trait03	0.3718	0.4708	0.5915	0.0000	0.0285	0.4506	0.2792	0.0000	2.2615	0.0877
Threitol/ Erytritol	Trait31	0.3816	0.5366	0.3527	0.2862	0.0195	0.4791	0.2016	0.0000	1.2939	0.0963
Internal standard	Trait63	0.4914	0.6659	0.5516	0.0020	0.0082	0.5484	0.0192	0.4827	0.9926	0.0794
Glycerol	Trait11	0.4107	0.3348	0.4929	0.0530	0.0285	0.5925	0.3166	0.0000	2.8339	0.0724
Spermidine	Trait27	0.0000	0.4592	0.6122	21.1734	0.0409	0.5999	0.4250	0.0000	1.2849	0.0873
Pentose alcohol IV	Trait72	0.6171	0.4338	0.5519	0.3801	0.0099	0.6199	0.1833	0.0000	0.6917	0.0738
Hexose II	Trait64	0.2123	0.3762	0.5101	0.0691	0.0128	0.6679	0.1937	0.0000	1.3286	0.0648
Maleic acid	Trait16	0.8510	0.5685	0.7043	3.1963	0.0121	0.8163	0.7355	0.0000	1.2909	0.0324
Benzoic acid I	Trait07	0.0000	0.4862	0.6248	0.0000	0.0000	1.0000	0.1512	0.0000	0.5308	0.0000
Nicotinic acid	Trait13	0.2566	0.2476	0.4350	0.0901	0.0000	1.0000	0.1259	0.0000	0.8687	0.0000
beta-Alanine	Trait26	0.1907	0.4752	0.6564	0.9625	0.0000	1.0000	0.0755	0.0036	2.3243	0.0000
5-Aminovaleric acid	Trait28	0.7019	0.4952	0.5197	2.2230	0.0000	1.0000	0.0931	0.0000	0.4798	0.0000
Malic acid	Trait29	0.4706	0.5920	0.5996	0.1600	0.0000	1.0000	0.1698	0.0000	0.6567	0.0000
4-Aminobutyric acid	Trait36	0.4892	0.5821	0.0000	0.0177	0.0000	1.0000	0.3294	0.0000	0.9798	0.0000
Threonic acid	Trait38	0.4413	0.7218	0.5556	6.0082	0.0000	1.0000	3.0232	0.0000	22.6880	0.0000
Lauric acid	Trait41	0.2897	0.3436	0.4928	0.4153	0.0000	1.0000	0.2926	0.0000	1.4516	0.0000
Gluconic acid 1.5-lactone	Trait52	0.4207	0.5125	0.6723	0.1364	0.0000	1.0000	0.0796	0.0000	0.5405	0.0000
Pentose II	Trait59	0.6178	0.4782	0.7705	0.7842	0.0000	1.0000	0.3072	0.0000	1.3436	0.0000
Hexose I (Glucose)	Trait61	0.1895	0.5651	0.0000	0.0698	0.0000	1.0000	0.2573	0.0000	0.8595	0.0000
D-Galactose II	Trait65	0.6142	0.4806	0.6368	0.0000	0.0000	1.0000	0.3338	0.0000	1.0115	0.0000
Hexose alcohol (sorbitol)	Trait71	0.2350	0.6095	0.4906	0.0679	0.0000	1.0000	0.3155	0.0000	1.4993	0.0000
Hexose (Glucose II)	Trait73	0.6944	0.5050	0.3879	0.9544	0.0000	1.0000	0.2094	0.0000	0.7102	0.0000
trans-caffeic acid	Trait76	0.4998	0.3571	0.6269	0.0485	0.0000	1.0000	0.4647	0.0000	1.9696	0.0000
Octadecanoic acid	Trait78	0.4083	0.4283	0.6652	6.6236	0.0000	1.0000	0.3814	0.0000	2.6144	0.0000
Oligo I (sucrose)	Trait80	0.5112	0.8408	0.6476	0.0000	0.0000	1.0000	0.1244	0.0000	0.4095	0.0000

Supplemental Table 3: Pairwise correlation coefficients and associated P values among the measured metabolites. P values are represented as: *** for P < 0.001, ** for P < 0.01, and * for P < 0.05. Data are sorted according to Fig. 2.

	Trait1	Trait2	Trait3	Trait4	Trait5	Trait6	Trait7	Trait8	Trait9	Trait10	Trait11	Trait13	Trait14	Trait15	Trait16	Trait17	Trait18	Trait19	Trait20	Trait21	Trait22	Trait23	Trait24	Trait25	Trait26	Trait27	Trait28	Trait29	Trait30	Trait31	Trait33	Trait34	Trait36	Trait37	Trait38	Trait39	Trait40	Trait41	Trait43	Trait44	Trait45	Trait46	Trait47	Trait48	Trait49	Trait50	Trait51	Trait52	Trait53	Trait54	Trait55	Trait56	Trait57	Trait58	Trait59	Trait60	Trait61	Trait62	Trait63	Trait64	Trait65	Trait66	Trait67	Trait68	Trait69	Trait70	Trait71	Trait72	Trait73	Trait75	Trait76	Trait77	Trait78	Trait80	Trait84	Trait85
Trait1		*	***	***	***	*	***	***					***		***	***	***	***		***	***	**						**				**	**		*		***	**			***		**	*									***	***			***	***	***	***	*	***			**		***						*			
Trait2	0.20		*	***	*	***		**	***	**	*	**	***	***		*	***	*	*											***	***	***	*			***	***	*		***				*	*		***			*	**	**				*		***	**		*	***						*	**	***			***	**		
Trait3	0.32	-0.20					***				*				***		*				**		*							**		*			**					**		*	*				***		*	*		***	***	*					*													*				
Trait4	0.47	0.39	-0.13		***	***		***		***	**	*	***	***		***	***	***		***	***		*	**			***	*		***	***	***	**	*		***	***		*	**	**		*	***	**	***	***			***	***	***	**	*			*	***	***			***		**	***	**	*						*			
Trait5	0.44	0.21	0.02	0.75		*		***		***			***	***	**	***	***	***	*	***	***	*	*	**			***	***		***	***	***	**			***	***	*	***		***	*	*	***	**	***	***	***		***	***	***	***	***	**	***	***	***	***	**	***	***		***	***	***	*	*		***				***		
Trait6	0.21	0.32	-0.17	0.48	0.66			***		***		**	***	***		***	***	***		***	***	***						**	**	***	***	***	***			***	***		***		***	**		***	***	***	***	***		***	***	***	**	***	***	***	***	**	***	**	*	***	**	***	***	***		***		***			***			***
Trait7	0.38	0.08	0.38	0.03	-0.02	0.08					*						***				*					***									**							*											**	*			**	**	*			*					**							*		
Trait8	0.46	0.24	0.12	0.37	0.55	0.50	0.16			***	**		***	***		***	***	***		**	***	*						**		***	**	**	***			**	***				**			***		**		**		*		*	**	**			***	***	***	*		***			**		*						***			*
Trait9	-0.03	0.30	-0.13	0.17	0.14	0.15	-0.03	0.06		***		***			*		**	**	***				*								***			**		**	***	***				***	**		***	*	***		***	***	***	***					***							***		***		***	**						**	
Trait10	0.09	0.27	-0.03	0.40	0.51	0.79	0.01	0.35	0.31			**	***	***		***	***	***		**	**	*								**	***	***	*			***	***		***		**			***	***	***	***	***	***	***	***	***	**	**	**	***		***	***	**		***	**	***	***	****				**		*	**			
Trait11	0.04	-0.22	0.19	-0.24	-0.07	-0.09	0.18	0.22	-0.09	-0.11					*																	*			**		**	*		**																			*				*													
Trait13	0.06	0.23	-0.07	0.18	0.09	0.27	0.07	0.06	0.37	0.28	-0.06		*	*	**			*								*					***	*				**	*					*	*	**	***	**	***		*	**	***	***														**							*	*		
Trait14	0.32	0.30	-0.03	0.55	0.69	0.80	0.05	0.49	0.13	0.86	-0.10	0.18		***	*	***	***	***		***	***	**	*	***			**	***		***	***	***	**			***	***	*	***		***		*	***	*	***	**	***	**	***	***	***	***	***	***	**	***	***	***	***		***	**	***	***	***		***	*	***		*	**	***		
Trait15	0.16	0.32	-0.05	0.43	0.32	0.48	-0.08	0.41	0.10	0.43	-0.07	0.21	0.43			***	**	***		**	*									**	***	**	**	*		***	***				*			***		***	***					*						***	***	*		***											***			
Trait16	0.44	-0.07	0.46	0.10	0.24	-0.11	0.12	0.13	-0.20	-0.01	0.14	-0.23	0.18	0.06		*	***	**	*	***	**		**	***			*	***							**			**			***		***		*		***			*	*	**	***	***			**	***	***	**	**	***					*		**					***		*
Trait17	0.29	0.22	0.10	0.39	0.39	0.50	0.13	0.48	0.12	0.44	-0.04	0.09	0.51	0.63	0.19		***	***		***	***	*						***		***	***	***	**	*		***	***		*		***		*	***		***	**	**	*		*	**	**	**	***	*	***	***	***	***		***	*	*	***	**	*	***					*			
Trait18	0.59	0.37	0.17	0.48	0.54	0.45	0.35	0.49	0.27	0.40	0.03	0.17	0.59	0.24	0.39	0.36		***		***	***			***				***		***	***	***	***		**	***	***	*	*		***			***	***	***	*	*		***	***	***	***	***			***	***	***	***		***			***	**	*						*		*	
Trait19	0.40	0.22	-0.01	0.42	0.52	0.53	0.15	0.54	0.26	0.47	0.05	0.19	0.58	0.42	0.23	0.59	0.68			***	*			*				***		***	***	***	**		*	***	***	*	**		***			***	**	***	*	***		**	***	***	***	***	**	**	***	***	***	***		***	*		***	***	*	**		*			*	**		
Trait20	-0.06	-0.18	0.01	0.02	0.22	0.17	0.04	0.00	0.30	0.16	0.09	0.12	0.03	-0.01	-0.18	-0.03	0.03	0.05													***								***			***			***		***	**	*	***	***	**	**	*	*	***		***				*		***		***				**	***			*		
Trait21	0.35	0.03	0.14	0.38	0.41	0.30	0.07	0.23	0.07	0.27	-0.04	0.06	0.30	0.24	0.46	0.34	0.39	0.46	-0.03			*					**	***		***	***	***			*	***	***		***		***			***	*	***		*		**	***	**	***	***			*	**	***	**	**	***		***	***	**	*	**		*				*	*	
Trait22	0.34	0.16	0.23	0.37	0.43	0.30	0.18	0.33	-0.16	0.27	-0.03	0.02	0.44	0.22	0.23	0.34	0.34	0.21	0.05	0.14		*	***	***				**		***	*	***	***			**	***				***			***				***		**	*		***	**	***		***	***	***	***		***		**	***	**	*	*	***					*		
Trait23	0.26	0.14	-0.07	0.16	0.19	0.36	-0.02	0.17	-0.04	0.18	-0.11	0.12	0.24	0.16	-0.09	0.20	0.04	0.13	0.01	0.19	0.18			**	**				***		***	***	*			***	***	**	*	***	*			***	**	***	**	**	*					*			*								*			***		**	*					**
Trait24	0.15	-0.08	0.21	0.20	0.18	0.10	0.03	-0.13	-0.20	0.11	-0.13	-0.10	0.18	0.12	0.27	0.06	0.10	-0.02	-0.05	0.13	0.32	0.06									*	*			*	*	**		**		***		**	**		*		***					**	***			*	*	***	***		*	*	*	***			**	*							
Trait25	-0.16	-0.05	-0.02	-0.22	-0.27	0.01	-0.14	-0.16	0.12	0.02	0.00	0.13	-0.28	0.00	-0.33	-0.09	-0.36	-0.21	0.16	-0.05	-0.31	0.25	-0.04					***	***	***	*							*					*		**	*	**						**		*		**	***	***			***					*		***		***	*		**		*
Trait26	-0.09	-0.04	0.09	-0.04	0.04	0.04	0.00	-0.08	-0.05	0.09	0.15	0.04	0.06	0.02	0.07	0.10	-0.13	-0.12	0.06	0.14	0.16	0.24	0.11	0.12			***		**	*	**					**		**	**			**		***		*			***	**	*		*	**	**						**			***				**	*	**			**			
Trait27	-0.06	-0.04	-0.04	-0.02	0.02	-0.02	-0.29	-0.02	-0.03	-0.02	0.12	-0.21	0.01	0.01	0.01	-0.08	-0.13	-0.07	-0.12	0.02	-0.04	0.12	0.06	0.06	0.09		***				*		**	***														**							*			**	*		*	*					***	*	**						*	***
Trait28	0.07	-0.10	0.05	0.29	0.33	0.14	-0.08	0.05	-0.10	0.12	0.09	-0.03	0.24	0.08	0.17	0.11	0.12	0.13	-0.05	0.28	0.09	0.13	0.21	-0.09	0.33	0.52		*		***		*			*	***	**		*		**		*	***		***		**					*	***	**	*					*	*		*				***	***	***		**	**	**		
Trait29	0.22	-0.06	-0.03	0.20	0.32	0.26	0.01	0.24	0.00	0.15	0.04	0.08	0.35	0.15	0.34	0.38	0.46	0.52	0.05	0.31	0.24	0.00	0.14	-0.33	-0.03	-0.06	0.20			***	***	***			*	***	***	**	**		***	**		***		***		***		*		**	*	***	***	***	***	***	***	***	**	***	**		**		***	***	***	***				***	*	
Trait30	0.07	0.02	-0.07	0.05	0.14	0.27	-0.12	0.06	0.01	0.13	-0.07	0.08	0.11	0.07	-0.10	0.11	-0.10	-0.01	0.16	0.16	-0.02	0.81	0.00	0.36	0.28	0.15	0.12	0.00			***					***	*	*	*	***		***		***		***	***	*	*		*					*		*			*			*				***		***	***					*
Trait31	0.17	0.35	-0.23	0.53	0.50	0.37	-0.01	0.34	0.07	0.27	-0.15	0.02	0.46	0.23	0.08	0.34	0.42	0.39	-0.10	0.36	0.39	0.14	-0.05	-0.45	0.18	-0.02	0.29	0.40	0.02		***	***	**			***	***		***	**	***		*	***		**	*			***	**	***	***	**	***		*	***	***		*	***	**	**	***	*	***	**	*		**					
Trait33	0.13	0.29	-0.06	0.43	0.49	0.66	0.11	0.26	0.38	0.53	-0.06	0.39	0.47	0.36	-0.12	0.43	0.44	0.42	0.34	0.38	0.18	0.40	0.18	0.21	0.23	-0.21	0.11	0.30	0.42	0.33		***	*		***	***	***	*	***	**	***	***		***	***	***	***	***	***	***	***	***	***	***	**	***	**	*	***	**	**	***		***	***	***		**		**	*					
Trait34	0.23	0.37	-0.17	0.51	0.45	0.47	-0.05	0.27	0.04	0.29	-0.17	0.18	0.42	0.26	-0.04	0.37	0.33	0.34	-0.12	0.37	0.37	0.30	0.18	-0.15	0.13	-0.02	0.17	0.46	0.16	0.61	0.50		***			***	***		*		**			***	**	***	***	***		***	***	***	**	**	**		*	***	***	*		***		**	***	**	***	***	*	*			*			
Trait36	0.23	0.18	0.07	0.28	0.25	0.29	-0.06	0.47	0.11	0.18	0.02	0.05	0.23	0.28	-0.02	0.24	0.31	0.25	-0.09	0.16	0.29	0.20	0.05	0.10	0.07	0.26	0.12	0.16	0.12	0.28	0.21	0.39		***		***	***				*			**	*	*		***			*		*	**	**			**	***	**		**			**			*					**			*
Trait37	-0.01	-0.12	0.16	-0.17	0.08	0.02	-0.05	0.14	0.25	0.01	0.11	-0.05	-0.03	-0.17	0.00	-0.18	0.02	0.01	0.12	-0.10	-0.06	0.09	-0.02	0.14	0.09	0.37	0.14	-0.10	0.09	-0.15	-0.05	-0.21	0.41															*										*	*			**														
Trait38	0.22	-0.10	0.26	-0.03	0.07	0.09	0.26	0.05	-0.08	-0.07	0.26	0.08	-0.01	0.03	0.26	0.08	0.25	0.18	-0.01	0.18	-0.16	0.10	0.17	0.15	0.12	-0.03	0.18	0.22	0.13	-0.16	0.29	0.07	0.02	0.00		**		**			**			**		***	*						**	***			**				***							**		**					*	
Trait39	0.13	0.40	-0.15	0.47	0.44	0.57	0.04	0.28	0.24	0.42	-0.17	0.25	0.45	0.37	-0.10	0.42	0.36	0.37	0.05	0.42	0.24	0.43	0.22	0.16	0.25	0.05	0.31	0.29	0.43	0.46	0.78	0.65	0.36	-0.03	0.24		***		***	**	***	***		***	***	***	***	***	***	***	***	***	***	***	*	**		**	***	*		***		***	***	***		***		*						
Trait40	0.28	0.45	-0.07	0.69	0.70	0.71	0.02	0.42	0.30	0.61	-0.23	0.21	0.70	0.48	0.09	0.54	0.61	0.55	0.07	0.43	0.41	0.29	0.27	-0.14	0.05	0.00	0.26	0.43	0.22	0.57	0.73	0.69	0.37	-0.06	0.16	0.80			***	*	***	**		***	***	***	***	***	**	***	***	***	***	***	***	**	**	***	***	***		***		***	***	***		**		**			*			
Trait41	0.27	-0.19	0.08	0.05	0.22	0.17	0.09	0.15	-0.28	0.12	0.22	0.06	0.20	0.02	0.22	0.08	0.17	0.21	0.04	0.16	0.14	0.26	0.06	-0.18	0.22	-0.13	0.12	0.26	0.21	0.16	0.19	0.07	-0.13	-0.15	0.24	0.01	0.02		***		***		**	***		**							***	**	**	**	***		*	***	***	**	***		*		***	**	**	***				**		
Trait43	0.02	-0.07	-0.09	0.17	0.44	0.52	-0.04	0.14	0.08	0.46	0.00	-0.04	0.44	0.13	-0.02	0.20	0.18	0.26	0.30	0.33	0.11	0.19	0.27	0.02	0.24	0.00	0.21	0.23	0.20	0.29	0.54	0.21	0.06	0.07	0.12	0.41	0.39	0.38			***	***	*	***	*	***	***	***	*	***	***	***	***	***	***	***	***		**	***	***	*	***	***	***	***	*	**		***		*		***		
Trait44	0.09	0.39	-0.26	0.23	0.10	0.17	-0.05	0.04	0.17	0.05	-0.22	0.13	0.10	0.05	-0.16	-0.01	0.09	-0.02	-0.02	0.01	-0.06	0.46	-0.10	0.03	0.08	0.02	0.00	-0.04	0.54	0.24	0.28	0.16	0.13	0.03	-0.07	0.27	0.20	0.00	0.04			***		*			***															*														
Trait45	0.30	0.04	0.11	0.27	0.43	0.38	0.09	0.28	-0.03	0.27	0.06	-0.06	0.38	0.19	0.29	0.30	0.36	0.37	0.03	0.36	0.33	0.22	0.39	-0.15	0.11	0.01	0.24	0.33	0.14	0.34	0.42	0.23	0.17	0.03	0.23	0.32	0.41	0.42	0.62	-0.03			***	***		***		***		*			***	***	***	***	***	**	***	***	**	***	***	**	***	*	**	**		**				**		
Trait46	-0.11	-0.02	-0.18	0.11	0.21	0.22	-0.19	-0.11	0.31	0.17	-0.03	0.19	0.11	0.07	-0.09	0.04	0.00	0.02	0.45	0.14	-0.07	0.13	-0.02	0.10	0.26	0.10	0.14	0.23	0.34	0.07	0.41	0.06	-0.06	0.08	0.06	0.29	0.27	0.12	0.35	0.38	0.05			***		***	***	*	***	***	***	**			*	***		*		*	*			***		***				***	*		*	**		
Trait47	0.25	0.05	0.18	0.18	0.22	0.11	0.00	0.15	-0.27	0.07	0.01	-0.21	0.20	0.13	0.30	0.20	0.10	0.08	-0.14	0.06	0.17	0.06	0.27	-0.19	0.07	0.03	0.20	0.05	0.05	0.18	-0.02	0.02	0.06	0.04	0.04	-0.01	0.12	0.26	0.21	0.08	0.32	0.03			***						**	***					**	***	***	**		***	***				**									*
Trait48	0.20	0.21	-0.09	0.48	0.52	0.68	0.07	0.33	-0.02	0.56	0.02	0.25	0.56	0.39	0.00	0.49	0.33	0.41	0.13	0.39	0.35	0.36	0.26	0.12	0.29	-0.03	0.28	0.39	0.35	0.39	0.71	0.55	0.26	-0.16	0.22	0.65	0.66	0.31	0.56	0.18	0.48	0.32	0.13		**	***	***	***	***	***	***	***	***	***	***	***	***	**	***	***	***	***	***	***	***	***	***	***		***						
Trait49	0.14	0.22	-0.11	0.27	0.22	0.38	0.12	0.05	0.43	0.31	-0.07	0.33	0.22	0.14	-0.21	0.10	0.33	0.27	0.29	0.19	0.04	0.23	0.11	0.27	0.02	-0.09	0.00	-0.02	0.15	0.05	0.55	0.26	0.18	0.01	0.15	0.46	0.42	-0.07	0.21	-0.02	0.14	0.08	-0.33	0.26		***	***		*	***	***	***	***	***										***		***										
Trait50	0.12	0.14	-0.11	0.31	0.47	0.64	-0.04	0.25	0.17	0.53	0.07	0.24	0.50	0.32	0.02	0.37	0.40	0.53	0.16	0.46	0.09	0.36	0.20	0.19	0.21	0.09	0.33	0.36	0.37	0.27	0.73	0.41	0.21	0.02	0.36	0.65	0.61	0.25	0.53	0.12	0.41	0.35	0.00	0.75	0.46		***	***	***	***	***	***	***	***	***	***	**		**	**	**	**	**	***	***	***		***		***						
Trait51	-0.12	0.41	-0.33	0.35	0.26	0.46	-0.09	0.01	0.54	0.38	-0.14	0.35	0.25	0.30	-0.32	0.26	0.18	0.19	0.32	0.12	-0.09	0.25	-0.01	0.27	0.15	0.00	0.13	0.15	0.33	0.22	0.71	0.33	0.11	-0.03	0.18	0.61	0.57	-0.09	0.33	0.41	0.11	0.60	-0.08	0.47	0.50	0.53		*	***	***	***	***				**								***		***			*							
Trait52	0.13	-0.04	0.13	0.13	0.36	0.41	0.00	0.23	0.09	0.39	0.08	0.10	0.39	0.07	0.07	0.25	0.19	0.31	0.22	0.17	0.42	0.24	0.31	0.11	0.11	0.24	0.25	0.43	0.20	0.13	0.33	0.36	0.31	0.19	0.12	0.36	0.40	0.10	0.38	-0.08	0.33	0.20	0.04	0.50	0.17	0.45	0.17		**	***	***	***	***	***	***	***	**		**	***			**	***	***	***		***	**	***	**			***		*
Trait53	0.04	-0.01	-0.17	0.09	0.15	0.17	-0.07	-0.01	0.34	0.28	-0.03	0.22	0.23	-0.01	-0.04	0.17	0.08	0.06	0.19	0.15	0.03	0.17	-0.02	0.15	0.34	-0.01	0.15	0.13	0.21	0.11	0.33	0.12	-0.02	0.01	0.04	0.30	0.24	-0.01	0.21	0.15	0.02	0.40	-0.12	0.35	0.18	0.30	0.31	0.26		***	***	***		*		*					*			***		***							*	*		
Trait54	0.07	0.17	-0.18	0.41	0.50	0.42	0.03	0.20	0.38	0.38	-0.09	0.23	0.38	0.05	-0.19	0.16	0.33	0.26	0.36	0.25	0.27	0.14	0.00	-0.02	0.23	-0.10	0.11	0.18	0.12	0.46	0.57	0.39	0.16	-0.05	0.00	0.50	0.53	0.11	0.48	0.11	0.20	0.34	-0.16	0.45	0.39	0.42	0.45	0.36	0.38		***	***	***	***	**	***	*		**		**	**		***	***	***				***				*		
Trait55	0.10	0.23	-0.13	0.38	0.44	0.47	0.14	0.16	0.52	0.43	-0.07	0.32	0.36	0.09	-0.18	0.19	0.38	0.33	0.45	0.30	0.22	0.16	0.02	0.11	0.18	-0.06	0.04	0.16	0.17	0.28	0.68	0.40	0.18	0.01	0.09	0.58	0.58	-0.02	0.37	0.09	0.16	0.36	-0.25	0.47	0.60	0.51	0.55	0.40	0.43	0.81		***	***	***	**	***			**			*		***	***	***				**	*					
Trait56	0.02	0.28	-0.30	0.39	0.45	0.51	0.01	0.22	0.52	0.47	-0.11	0.34	0.43	0.18	-0.23	0.26	0.38	0.41	0.23	0.27	0.10	0.16	-0.10	-0.03	0.06	-0.13	0.03	0.27	0.13	0.40	0.62	0.48	0.16	-0.05	0.06	0.56	0.59	0.02	0.33	0.14	0.15	0.23	-0.36	0.45	0.50	0.48	0.58	0.28	0.40	0.68	0.74		**	***		*			*			**		***	*	***	*			**						
Trait57	0.41	0.01	0.31	0.23	0.42	0.24	0.27	0.26	0.13	0.24	0.06	0.06	0.34	0.11	0.37	0.27	0.59	0.56	0.24	0.48	0.36	0.03	0.26	-0.23	0.17	-0.07	0.21	0.41	-0.03	0.30	0.41	0.25	0.18	0.06	0.25	0.33	0.40	0.29	0.37	-0.13	0.48	0.09	0.08	0.29	0.33	0.38	0.06	0.34	0.09	0.34	0.40	0.26		***	***	***	***	***	***	***	*	***	***	***	***	**	**	*		**				*		
Trait58	0.35	0.02	0.22	0.18	0.41	0.31	0.21	0.28	0.15	0.27	0.12	0.07	0.34	0.13	0.29	0.28	0.49	0.55	0.17	0.43	0.28	0.18	0.29	-0.07	0.25	0.06	0.30	0.36	0.09	0.27	0.46	0.28	0.28	0.17	0.30	0.42	0.42	0.27	0.41	-0.11	0.54	0.07	0.08	0.38	0.41	0.50	0.15	0.40	0.19	0.32	0.40	0.29	0.88		***	***	**	**	***	***	*	***	***	***	***	**		**		**				*		
Trait59	-0.09	-0.12	-0.05	0.15	0.27	0.32	-0.10	0.15	-0.07	0.28	0.09	0.07	0.36	0.16	0.05	0.34	0.12	0.24	0.18	0.09	0.41	0.10	0.13	-0.19	0.28	0.21	0.25	0.43	0.13	0.34	0.24	0.27	0.23	0.00	-0.13	0.21	0.30	0.24	0.30	-0.08	0.34	0.18	0.06	0.40	0.08	0.31	0.10	0.45	0.10	0.27	0.22	0.16	0.31	0.32		***			**	***		**	***	***	***	**		***	***	***				**	*	*
Trait60	-0.04	-0.20	-0.04	0.04	0.29	0.40	0.02	0.12	0.14	0.32	0.14	0.08	0.25	0.10	-0.12	0.20	0.11	0.24	0.47	0.16	0.12	0.11	0.07	0.08	0.17	0.14	0.18	0.33	0.21	0.14	0.39	0.12	0.13	0.13	0.06	0.24	0.26	0.23	0.63	0.05	0.35	0.38	0.02	0.47	0.16	0.44	0.25	0.49	0.18	0.35	0.36	0.21	0.30	0.32	0.52		***		*	***	*		***	***	**	***		***		***	**	*		***		
Trait61	0.40	-0.06	0.12	0.20	0.43	0.32	0.25	0.36	-0.30	0.09	0.13	-0.11	0.31	0.11	0.24	0.32	0.28	0.34	0.03	0.20	0.38	0.21	0.22	-0.23	0.09	-0.15	0.11	0.33	0.13	0.19	0.24	0.21	0.01	-0.10	0.25	0.16	0.24	0.52	0.38	0.01	0.41	0.07	0.27	0.42	-0.08	0.27	-0.03	0.25	-0.05	0.21	0.08	0.09	0.31	0.27	0.15	0.34		**	***	***	***	***	**				***	***	*	***	*			***		
Trait62	0.48	0.33	0.10	0.54	0.37	0.27	0.26	0.40	0.04	0.30	-0.14	0.05	0.52	0.32	0.34	0.49	0.66	0.48	-0.28	0.27	0.46	0.01	0.21	-0.53	-0.05	-0.23	0.03	0.39	-0.17	0.53	0.22	0.45	0.22	-0.21	-0.03	0.27	0.50	0.16	0.05	0.10	0.28	-0.19	0.33	0.28	-0.01	0.10	-0.04	0.02	-0.01	0.12	0.09	0.16	0.34	0.24	0.11	-0.12	0.26		***	**		***			***		***		**		**		*		*	*
Trait63	0.45	0.23	0.21	0.58	0.50	0.41	0.21	0.42	0.01	0.41	-0.18	0.11	0.57	0.36	0.32	0.45	0.63	0.49	-0.05	0.41	0.52	0.02	0.30	-0.44	-0.01	-0.18	0.05	0.41	-0.11	0.53	0.36	0.51	0.31	-0.18	-0.01	0.33	0.58	0.22	0.28	0.11	0.39	-0.01	0.33	0.46	0.02	0.26	0.02	0.23	-0.01	0.28	0.27	0.20	0.49	0.34	0.22	0.17	0.34	0.84		***		***	**	**	***	*	***	*	**				**			
Trait64	0.29	-0.08	0.15	0.17	0.24	0.24	0.07	0.19	-0.07	0.27	0.08	0.00	0.34	0.21	0.27	0.36	0.31	0.28	0.13	0.22	0.35	0.13	0.33	-0.14	0.05	0.11	0.14	0.47	0.08	0.14	0.23	0.22	0.24	0.00	0.11	0.19	0.31	0.29	0.44	-0.02	0.46	0.17	0.25	0.42	0.03	0.28	0.04	0.50	0.08	0.10	0.15	0.03	0.45	0.41	0.46	0.57	0.35	0.27	0.45			**	***	***	***	***	*	***	***	***	*			**		
Trait65	0.22	-0.21	0.02	0.11	0.34	0.17	0.11	0.16	-0.15	0.05	0.04	-0.06	0.15	-0.07	0.27	0.13	0.14	0.11	0.05	0.27	0.03	0.11	0.02	0.02	0.28	-0.19	0.22	0.27	0.20	0.21	0.22	0.10	-0.07	-0.03	0.32	0.17	0.12	0.37	0.37	0.06	0.24	0.20	0.13	0.32	-0.09	0.28	0.06	0.13	0.22	0.26	0.08	0.09	0.18	0.18	0.02	0.17	0.46	0.08	0.12	0.07		*	*	**			***	***		***			***	***		
Trait66	0.39	0.34	0.05	0.62	0.55	0.41	0.21	0.44	-0.08	0.34	-0.10	0.10	0.58	0.36	0.30	0.48	0.63	0.50	-0.21	0.33	0.48	0.03	0.19	-0.56	0.08	-0.18	0.20	0.42	-0.12	0.69	0.34	0.55	0.24	-0.24	0.06	0.39	0.59	0.24	0.20	0.18	0.33	-0.09	0.32	0.43	-0.02	0.28	0.07	0.05	-0.01	0.26	0.17	0.26	0.39	0.30	0.23	0.00	0.35	0.85	0.81	0.24	0.21		*		***		***	**	**		*		*			
Trait67	0.08	-0.11	-0.02	-0.01	0.16	0.25	0.06	0.12	-0.04	0.23	0.18	-0.11	0.24	0.03	0.14	0.17	0.16	0.19	0.08	0.12	0.11	0.10	0.19	-0.16	0.14	0.09	0.17	0.22	0.08	0.23	0.20	0.01	0.06	0.04	0.11	0.07	0.11	0.51	0.62	0.00	0.68	0.11	0.41	0.30	-0.02	0.24	0.05	0.26	0.03	0.12	0.06	0.01	0.29	0.38	0.37	0.50	0.28	0.13	0.24	0.51	0.17	0.17		**	*	*	**	*		**		*		**		
Trait68	0.01	-0.02	0.07	0.27	0.46	0.47	-0.05	0.10	0.32	0.65	-0.09	0.16	0.54	0.14	-0.02	0.20	0.16	0.15	0.36	0.30	0.27	0.16	0.17	0.08	0.35	0.05	0.20	0.08	0.20	0.26	0.53	0.25	0.16	0.08	-0.02	0.39	0.46	0.13	0.61	0.02	0.27	0.39	0.02	0.44	0.29	0.39	0.38	0.46	0.47	0.60	0.55	0.45	0.32	0.30	0.29	0.44	0.12	0.02	0.27	0.30	0.23	0.12	0.27		***	***				***		**		***		
Trait69	0.23	0.01	0.15	0.31	0.42	0.36	-0.01	0.27	0.05	0.42	-0.13	0.09	0.44	0.16	0.14	0.31	0.30	0.35	0.12	0.31	0.50	0.21	0.28	-0.10	0.17	-0.01	0.13	0.25	0.10	0.30	0.32	0.36	0.23	0.04	-0.09	0.30	0.43	0.18	0.33	-0.02	0.35	0.04	0.13	0.36	0.07	0.31	-0.02	0.37	0.12	0.30	0.30	0.20	0.43	0.40	0.38	0.27	0.14	0.34	0.48	0.37	0.11	0.38	0.21	0.43		***				*	*			*		
Trait70	-0.01	0.17	-0.09	0.23	0.36	0.46	0.03	0.12	0.45	0.60	-0.08	0.27	0.45	0.14	-0.11	0.27	0.26	0.29	0.33	0.23	0.23	0.11	0.02	0.09	0.09	-0.14	-0.03	0.08	0.12	0.21	0.54	0.23	0.04	-0.02	-0.09	0.39	0.50	0.02	0.44	0.11	0.19	0.33	-0.05	0.44	0.38	0.40	0.45	0.33	0.48	0.55	0.64	0.59	0.25	0.26	0.24	0.42	0.07	0.05	0.18	0.29	0.04	0.13	0.20	0.67	0.37				**		*			*		
Trait71	0.32	-0.04	-0.08	0.20	0.21	0.10	0.22	0.17	-0.10	-0.02	0.00	0.05	0.11	-0.05	0.21	0.20	0.21	0.20	-0.12	0.19	0.20	0.10	0.06	-0.22	0.10	-0.40	-0.05	0.35	0.03	0.34	0.17	0.33	0.01	-0.16	0.05	0.10	0.10	0.38	0.17	0.10	0.27	-0.08	0.22	0.29	-0.05	0.06	-0.11	0.05	0.11	0.16	0.07	0.17	0.23	0.17	0.04	0.04	0.39	0.38	0.34	0.21	0.39	0.40	0.24	0.02	0.13	0.13										
Trait72	0.02	-0.19	-0.08	0.10	0.21	0.29	-0.16	0.11	-0.30	0.13	0.00	0.07	0.29	0.16	0.07	0.32	0.05	0.22	-0.08	0.22	0.18	0.30	0.23	-0.07	0.24	0.18	0.39	0.42	0.31	0.23	0.23	0.37	0.17	-0.07	0.27	0.33	0.27	0.25	0.26	-0.01	0.26	0.08	0.11	0.45	-0.02	0.49	0.08	0.36	0.13	0.09	0.05	0.06	0.17	0.23	0.44	0.32	0.36	0.12	0.22	0.33	0.30	0.28	0.20	0.10	0.12	-0.08	0.10		***	***				*		
Trait73	0.12	-0.28	0.13	0.03	0.08	0.00	-0.10	0.03	-0.27	-0.02	0.02	-0.08	0.19	0.00	0.26	0.15	0.06	0.07	-0.16	0.16	0.29	0.11	0.17	-0.37	0.19	0.26	0.32	0.32	0.03	0.19	-0.17	0.21	0.15	0.08	0.04	0.02	0.06	0.24	0.07	-0.11	0.14	0.04	0.16	0.06	-0.17	0.03	-0.19	0.26	0.03	-0.06	-0.12	-0.14	0.16	0.09	0.38	0.11	0.20	0.24	0.28	0.34	0.10	0.24	0.17	0.05	0.14	-0.23	0.05	0.62		***				**		
Trait75	-0.01	-0.31	0.01	0.04	0.36	0.35	-0.11	0.10	-0.09	0.23	-0.01	0.00	0.31	-0.02	0.04	0.13	0.08	0.21	0.24	0.20	0.06	0.23	0.11	-0.03	0.25	0.11	0.30	0.39	0.33	0.14	0.26	0.18	-0.01	0.08	0.26	0.22	0.25	0.33	0.47	-0.04	0.27	0.31	-0.07	0.41	0.08	0.48	0.15	0.43	0.14	0.31	0.24	0.25	0.25	0.26	0.40	0.55	0.41	-0.12	0.12	0.33	0.42	0.04	0.25	0.36	0.21	0.11	0.02	0.49	0.31			*		***		
Trait76	0.06	-0.14	0.14	-0.07	0.06	0.11	-0.02	-0.01	-0.06	0.03	-0.03	0.15	-0.04	0.07	-0.07	0.03	-0.11	0.02	0.37	0.04	0.11	0.21	0.11	0.33	0.01	0.03	-0.11	-0.01	0.35	-0.27	0.21	0.03	0.07	0.07	0.16	0.11	0.04	0.12	0.14	0.03	0.07	0.18	-0.04	0.16	0.11	0.14	0.06	0.22	0.00	0.08	0.19	-0.05	0.16	0.14	0.15	0.27	0.18	-0.24	-0.02	0.20	-0.02	-0.17	0.04	0.12	0.18	0.17	-0.11	0.16	0.03	0.16		*				*
Trait77	-0.05	-0.09	-0.18	0.08	0.06	0.09	-0.08	-0.03	-0.04	0.18	0.08	0.05	0.21	0.09	0.06	0.08	-0.06	0.07	-0.02	0.05	-0.12	-0.12	0.04	-0.18	0.13	0.11	0.25	0.14	-0.13	0.13	-0.03	0.04	-0.05	-0.06	0.04	0.02	0.04	0.15	0.18	-0.11	0.07	0.14	0.16	0.12	-0.04	0.07	0.12	-0.02	0.17	0.07	-0.02	0.03	0.03	0.07	0.17	0.20	0.15	0.00	0.00	0.13	0.10	0.11	0.21	0.25	-0.07	0.15	0.15	0.08	0.11	0.18	-0.18		*			*
Trait78	-0.17	-0.30	0.00	-0.17	-0.09	-0.32	0.00	-0.31	0.01	-0.24	0.01	-0.21	-0.23	-0.32	0.13	-0.19	-0.21	-0.19	0.12	-0.01	-0.14	-0.06	0.05	0.00	0.22	0.06	0.26	-0.03	0.09	-0.08	-0.12	-0.19	-0.25	0.08	0.08	-0.05	-0.21	0.11	0.06	-0.04	-0.04	0.21	-0.02	-0.15	-0.08	-0.09	0.00	-0.06	0.18	-0.04	-0.07	-0.11	0.03	0.06	-0.07	0.00	0.00	-0.20	-0.25	-0.07	0.29	-0.22	0.08	0.13	-0.08	-0.04	0.04	0.04	0.12	0.12	0.10	0.18		**		*
Trait80	0.14	-0.27	-0.09	0.07	0.39	0.15	-0.22	0.16	0.01	0.13	0.05	-0.20	0.31	0.00	0.29	0.12	0.13	0.24	0.20	0.21	0.21	0.05	-0.01	-0.25	0.05	0.15	0.26	0.32	0.05	0.15	0.00	0.00	-0.04	0.11	-0.04	0.00	0.14	0.24	0.37	-0.10	0.28	0.27	0.10	0.07	-0.15	0.09	-0.09	0.29	0.21	0.22	0.10	0.09	0.19	0.18	0.24	0.35	0.36	-0.02	0.09	0.26	0.31	0.02	0.28	0.33	0.23	0.20	0.06	0.19	0.26	0.40	0.06	0.14	0.24			
Trait84	0.06	0.10	-0.01	-0.08	-0.13	-0.14	0.10	0.01	0.23	-0.12	-0.04	0.08	-0.04	-0.06	0.00	0.05	0.17	0.10	-0.01	-0.20	-0.11	-0.13	0.09	-0.10	-0.14	-0.19	-0.01	0.18	-0.16	-0.05	0.01	-0.06	-0.05	0.00	0.17	-0.02	-0.01	-0.15	-0.12	-0.03	0.03	-0.09	0.09	-0.14	-0.04	-0.04	0.06	-0.02	0.14	-0.04	-0.02	-0.01	0.01	0.02	-0.20	-0.10	-0.01	0.21	0.08	-0.06	0.00	0.03	-0.01	-0.12	-0.04	-0.12	0.02	0.02	-0.03	-0.07	-0.14	0.05	0.15	-0.01		***
Trait85	-0.02	0.06	0.12	-0.04	-0.17	-0.28	0.09	-0.18	0.11	-0.17	0.04	-0.02	-0.16	-0.07	0.19	0.00	0.09	-0.11	-0.03	-0.02	-0.09	-0.28	0.13	-0.20	0.06	-0.29	-0.07	0.16	-0.19	0.07	0.05	0.00	-0.19	-0.15	0.14	0.00	0.02	-0.06	-0.10	-0.08	-0.10	0.09	0.20	-0.12	-0.08	-0.08	0.11	-0.22	0.10	-0.03	-0.01	-0.04	0.06	0.01	-0.21	-0.14	-0.04	0.20	0.11	-0.04	0.09	0.14	-0.01	-0.01	-0.06	-0.02	0.10	-0.16	-0.09	-0.16	-0.22	0.18	0.17	-0.11	0.47	

Supplemental Table 4: Sequences of SNPs for which significant associations to metabolic traits have been observed. SNP sites are indicated in bold within the sequence. Detected variations are shown and the corresponding amino acid variations are given in the one letter code parenthesized.

Metabolic Trait	Locus	Sequence	Variation of amino acid
Oxalic acid	IWB8786	CCGCGCGGAGGACGACGAGATCCGACACATGCTCGTGATTTCCGTCCAAC**[A/G]**CCTT GCATCCAATGTACAGAAGCGACACCGAATCGAGCTAAAACGGCAAG	
	IWB4387	ATGTGAGGCGGACCGGGCACCGACGCACGGGGCCGAGCAGCGAGGCTTTA**[T/C]**AGG GGAGCCGGAGCTGCAACGGGGAAGGCTCGGTGCTGCAATCATCAAAC	
	IWB14354	GGTATCGAAGCGGAAAGAACCTGACTCAGGTTGGGATATCTACCCCCGGC**[A/G]**CTGA GCGTCCAATGTTAGTTGCAGTTGTATTTCTTCGGTTAATTCAATGT	C**A**C(**H**) C**G**C(**R**)
	IWB35367	TCCGATCGATCGGCAAGACACCGCATGACACCATCTCATGCCTCGAGCTTGAGAATGAA GGGAAGACCAGACCAGGACAATCACACAGCATGAGCTCTTC**[A/G]**GAGATTATCAATG TCTGGAAATGTTTCGTCTTACCGGGTGTGTGAGTGACACCGGTCTTCTTCTCGCCAACTA AAGCATTTATCGTGGAACTCTTCCCGA	TC**T**(**S**) TC**C**(**S**)
	IWB47615	ATGCATCTTCTGGGCAGCTGTATTTTCTAGACGAAGCCTTGTTGTGTTAG**[T/C]**ATCGGA ACCCTGCATGCACGTACCCATGCTTATTTATGTATGTCTGTGTA	
	IWA3211	TGTGGAAATCCTAGAGCTTTGCTTCCGCGGAGCTGGTCCCAGTATTCTGGACCGGATTG GCTCTTGCTGCTTTTTTCAAGAATTTGCCAGGACAGAAGAG**[A/G]**ACTGGTTCTCATTTT GTGGAGAGGCTGGCACTTAAGGTGTCACCGAAGGAATCTAAGCCGTGAAATCTTGTAG GAGAGCCTCAAATACTATGTTACAATG	
	IWA6946	GTAGCGATGATTTCACCATCAAGGAATCCGACGGTCATGAGTGGGATGAGTATGGAGA GGAGTAGTATATATAGTGATGGTCTTCTGAAAGGAGGTGATA**[A/G]**AGCCAAGCTTGA GGAAGCGGTCAATCAGTTGATGGCGACATCGATGGTGACGTCGCTTGCTGGTAAGATA YTTGATGCTCCGGCTGGAGCCGACAGGGTG	
	IWA6947	TAGTGATGGTCTTCTGAAAGGAGGTGATARAGCCAAGCTTGAGGAAGCGGTCAATCAG TTGATGGCGACATCGATGGTGACGTCGCTTGCTGGTAAGATA**[T/C]**TTGATGCTCCGGC TGGAGCCGACAGGGTGCGTGGAGCTGGTGATCAAGGAGTCTTGCGTGTATTGAAATGC GAGCACCAGGAAAGCATGGCGACTGATCC	
	IWB29877	AAGAAGAAGGGCCTACATCATCATCAGGTACAGATGATAAAGACACTGGC**[A/G]**GTGA ATTTTGGTTGACAAGATCTGCGAAGAGCTTTGGGGATTCCTCAAAG	**G**GT(**G**) **A**GT(**S**)
	IWB33544	GTATCATTGAACGGCGAACTGCATGTGCTTAAATCTGCCAAACTTCCTGC**[T/C]**GAAACC CTCCATCCTCGGAGGCAGTTAAAGAAGAGGCTGGCTTTGGAGTT	GC**C**(**A**) GC**T**(**A**)
Ornithine	IWB4446	TACGACCTCGGAATCTCAGGCGGCGTCACGTCCATGGAGTCGTTCCTCAA**[T/G]**AAATT CTTCCCGGACGTGTACCACCAGATGAACGGCGACAAGGCCGCCGT	**G**AA(**E**) **T**AA(**stop**)
	IWB6885	CGCATAGTTGTTCGAATTAATTCAATGGAGCTGTAATTCACAAGGCCATC**[T/C]**TGTGTG TTGTAATTTTGTTTCGGTTGTATCTAGTTGTTCTGTCTGATATA	
	IWB8628	CGTCACTTGTTATCACGACAAACATCAAAAAGTATGTACCGTTTGGGCAT**[A/G]**AGAAG GGCAACCGTTTCAGCGCCGCCGTGCCAGAAAACACATGTGTTGTG	
	IWB8637	GGAGACCATCATATTGGATTCCAATCTTCCTCAAAGGTTTGGCTGCATTG**[T/C]**GGCTAC TGATTACGCCATATATGATGGCATTGGCCTCGACAAGAAGCTTG	
	IWB60850	TGGCAATGTCGACGAGGCGAAGATGATGCTCCAGAGGCTGCGTGGCACGA**[T/C]**GGG GGTTCAGAAAGAGCTCGACGATCTGGTCACTGCCAGCAACATATCCA	A**C**G(**T**) A**T**G(**M**)
	IWB75174	TCGTGTCGTGTGGTGATTATGCCGTTTGCGCTGTTTACGTGTAATTAACC**[A/G]**TACCGA CTGTAACATGTATCCTCTGATCACCTGTTAATTTGTGTATCCCA	
	IWB3615	AGCGATTTATCCATTCCAACGAACAGGCACCATACATCATAAATTACATC**[T/G]**ATCACC ATGAACACTGAACAACGCCTAGCTACTGCTAATCTGAATATCTG	
	IWB8638	ACAAGGTGTTGTAACCTTCAAGAAGATCCAAGAATTTGAAAACGGGAAAA**[T/G]**TAGG TTAAGGGAGCTTCAGATCCGTGCCGTGTGCGTCAAATTCTCGTCGT	
	IWB46787	CCTGAACCTGATTCATCATCTGTTCATGTTGAGATGGGATCTGAAACGGC**[A/G]**CCAGA AGATGGAGGGAAGGCGCCTCGCCCGAGCAAATGGAAAATAAGGAC	GC**G**(**A**) GC**A**(**A**)
	IWB48775	TCTCACCGGTGAACCTCCTGATGAAGCCGAAGAGAGGCCAGCAAAGGAAG**[A/C]**GTCG	**A**GT(**S**)

		GCAGAAGCGTGACTTCCAGAAAGACATCCTGCCTAGCATCTCGTCG	**C**GT(**R**)
	IWB58986	CAAGTCAGCAACCAATCTTTTTTTACACCCAGATACAGCAATGCTGATCT**[A/G]**CAAAAA<br>TCCTTCAAAGTTGCTGATGGGAGATCTGCAACCAATGAGATTAT	CT**G**(**L**)<br>CT**A**(**L**)
	IWA2098	AAAGTGTYGGAAGCACCCCTTGTTTGCTGGGAAAGGCCGTCGACTGCAGCTACATACGT<br>GCCCCGGGGTACTTGGAAGTGGATGTTGACATCGGTTCTTC**[T/C]**GCGGTAGCCAATG<br>GGGTTCTGGGGCTGGTGTTTGGTGTTGTCACAACATTGGTAGTTGACATGGCCTTCCTA<br>ATACAGGCGAACACGTACGAGGAGCTCC	TC**T**(**B**)<br>TC**C**(**S**)
	IWB55921	CTTCTTCCACTGGGCGTGAGCAACCTGAACAGACCAAATCTAGCACGAGC**[A/G]**CCTTG<br>CTCTCCTGACTCTTCTACCAGAACTTTGGAGCCAGAAGTTGAGAA	GC**A**(**A**)<br>GC**G**(**A**)
	IWA2558	ATCAAGTCCAGGAGTAACTGCCAGGTGAAGGCTCAAGAGAAGGCTAGCCATCTCAGGA<br>ATAGGTAATGGTTTTACACCTCTAACCACTTCTTGAACCTTG**[A/G]**GTGACTGAACATTC<br>CATGTTTTCCAATGGGCGAAAAAATCTTCAGGGGAAAGAGTTATAGGTTGCAAGAATTT<br>ATGCAACACAATAGGAAGTCGAAGTTT	C**C**C(**P**)<br>C**T**C(**L**)
	IWB5567	GTGACAGCGACGTACCCAGGCCACCGGGAAGTCATGGAGTACATCAGGTC**[A/G]**TACG<br>TGTGCGAGTTTGACCTCCTCAAGTGTATCAAGTTCAATAGTCAGGT	TC**A**(**S**)<br>TC**G**(**S**)
	IWB7364	ACTCATGGGTCATGGCATTCACCAACAAGCATGCTCTCTCCAAGGCAGCG**[T/C]**GTGCG<br>TGTTAGTGTGTGTGGATCACCACATTAATCAGCACCATAACCAGA	
	IWB43461	GACATCTTCACAGAAAACTGGTGCAGAAATATAGTGGCAGGATCATCAGA**[T/C]**ACATC<br>GGTTCCACTATACAGAGAATGCATCCATCCTCGAATCCCTCAGTT	GA**T**(**D**)<br>GA**C**(**D**)
L-Arginine	IWB56221	CAGCAGAGGATGTGTGGCGGACCGGCTTCATCCGGATCGACCTCGTAGGA**[A/G]**CAGC<br>CAGCTTCTTCAAGCCAGGAAAGTCCCAAGAAATGGTTATTGGGCAG	**3'UTR**
	IWB65729	ACTAGAGGTCACAGCCAAAGAACAAATAGCATAGGaTTATCAACATCCAC**[T/C]**ACC	**3'UTR**
Pentose<br>alcohol III	IWB242	TTGTATTTACAGATTGTGGACATCAAGAATTTACCATCTGCAATTCCTGA**[T/G]**AAAAGC<br>GTTTATGTATGGTACACCAAGACTAAACCAGATGCATTCATCCG	GA**G**(**E**)<br>GA**T**(**D**)
	IWB1705	CCAGCTGCAGCAGCTGCCGCAACTGGTGCCGCTCCTAGCAAGTATGTCCC**[A/G]**AAGT<br>GGAAACGTGAGGGTGATAGTAGCAGTCAGAGGCCGACTGCGGCAGC	CC**G**(**P**)<br>CC**A**(**P**)
	IWB25161	GCCATTGGATAGAGGATGGATCCAAATATCAACGGAAATGCTGCTCCAAT**[A/C]**GGAA<br>TCTCGGCCAGCAGCTTAGATGAGAGATATGGTCCTAGTGCATACGA	CC**T**(**P**)<br>CC**G**(**P**)
	IWB32797	AACCAAGAATTACTGGCTTCAAAGGAAGAAGGTTAAGAAACATTTCTTAA**[T/C]**AATTT<br>AGCATATTCTTGCTCTAGGATAACCATTATTGTTCGAGGTTGATT	
	IWB35825	GTAGAAAATATAAGTAATTCCGTATTACATGGTACCGTCAAGTGAATCACACAGGTATC<br>TAATCCATTTTTTCTTATACACGCGATGCTAATTACAACCA**[T/C]**CATATACAGTTTCCAA<br>TACCTAATTAACAGTGCATAGATAATTAGATATGTGAACCGCACACAACA	
	IWB56509	TTGTATCTAGTCTTGCACTGAGGACAACATTTGACCCCATCCTTGCGTTC**[A/G]**TACTCA<br>TAGCAAGGGCGGCAGACCGGGAAGGCGCACTCATTGCAGGCGAC	TA**T**(**Y**)<br>TA**C**(**Y**)
	IWB65026	GATTCTGATACAAATACTGATGCTCTAGCCAATCTGCCTGCTACCGACAA**[T/C]**GATCAA<br>AATAACCAGCATCCGGTTTCAGCAGGTCCTGCCAGCTTGTCCAT	AA**T**(**N**)<br>AA**C**(**N**)
	IWB35763	AGGACAAGCCATCAGATTCTGACATCCATAAGCGTCACAGGAGCCGCTCTATGTCCTTG<br>GATGATGGTGCTGCTGGTGAGCCAGACAAGATGAATGATGG**[T/C]**AAGGGGTCTCACA<br>AAAGCCGGCACCACCGCCGCCATCACCATCACCATCATGATCACCATGGCAACTCTGCT<br>GAACCCAGTGACGGGAAGCAACTCGTGT	GG**C**(**G**)<br>GG**T**(**G**)
	IWB18115	ATGATCAGGACTTACAATTTTTCTTTGTCAATAGTACATAGTCGAGGCCATAAGTAACTC<br>TGCACATCCTGCATGAGTGGATATCTTCAAGAAAGGAAAGTAGGCATAAGAACAGGGT<br>TGTCAA**[T/C]**CATAGTTAGTTATTCACATATTCAGGTGCAAAAAAATCTTACAGGTCAAC<br>CGAATGAAACTCCTCAGAACAGCATTGCAGTTCAACCCATTGTAGCCTTGTGACTATGC<br>GAGCTACGTTGACATC	**Intron**
L-Tyrosine	IWB49741	GATCCTAGATTGACAGGAAGCTTCTCTCTACTTGCTGTTTGTACCTTCTC**[A/G]**ATGCCC<br>AGCCAGACGGGGCCGACGTCAGCTTCTTTCTACATTCCCGACAC	
Oligo II	IWB6807	GGGAAAACTACATTCATCAGAATGCTGGCTGGGCACTTGAAGCCAGATAC**[A/C]**GTGG<br>ATGGAGTTGAGATCGAAATTCCTGAATTTAATGTGTCCTACAAGCC	AC**A**(**T**)<br>AC**C**(**T**)
